# Investigating Possible Enzymatic Degradation on Polymer Shells around Inorganic Nanoparticles

**DOI:** 10.3390/ijms20040935

**Published:** 2019-02-21

**Authors:** Lin Zhu, Beatriz Pelaz, Indranath Chakraborty, Wolfgang J. Parak

**Affiliations:** 1 Faculty of Physics, Universität Hamburg, 22761 Hamburg, Germany; lzhu@physnet.uni-hamburg.de (L.Z.); chemistry.indra@gmail.com (I.C.); 2 Centro Singular de Investigación en Química Biolóxica e Materiais Moleculares (CiQUS), Departamento de Física de Partículas, Universidade de Santiago de Compostela, 15782 Santiago de Compostela, Spain; beatriz.pelaz@usc.es; 3 CIC Biomagune, 20014 San Sebastian, Spain

**Keywords:** nanoparticles, surface engineering, enzymatic degradation, polymer coating, bioconjugation, click chemistry, nanoparticle degradation

## Abstract

Inorganic iron oxide nanoparticle cores as model systems for inorganic nanoparticles were coated with shells of amphiphilic polymers, to which organic fluorophores were linked with different conjugation chemistries, including 1-ethyl-3-(3-dimethylaminopropyl)carbodiimide (EDC) chemistry and two types of “click chemistry”. The nanoparticle-dye conjugates were exposed to different enzymes/enzyme mixtures in order to investigate potential enzymatic degradation of the fluorophore-modified polymer shell. The release of the dyes and polymer fragments upon enzymatic digestion was quantified by using fluorescence spectroscopy. The data indicate that enzymatic cleavage of the fluorophore-modified organic surface coating around the inorganic nanoparticles in fact depends on the used conjugation chemistry, together with the types of enzymes to which the nanoparticle-dye conjugates are exposed.

## 1. Introduction

After in vitro or in vivo administration, nanoparticles (NPs) may be exposed to different local environments along their trajectory. As an example, intravenously injected NPs would first be localized in blood, likely to be followed by uptake to the acidic endosomes/lysosomes of macrophages. Depending on the details of the uptake trajectory, the NPs will be surrounded by different local exposure conditions. An acidic pH, such as that present in endosomes/lysosomes, may, for example, degrade NPs [1,2]. Enzymes may digest parts of the surface coating of the NPs, which can modify their physicochemical properties and consequently also their biodistribution [3,4]. NPs in general are hybrid materials comprising different entities such as core materials, surface functionalization, and the corona of adsorbed biomolecules (particularly proteins) [5]. Specific enzymes may initiate enzymatic reactions of disparate parts of NPs [6]. Enzymatic degradation thus may be selective to specific parts of the NPs [7,8,9]. Proteases present in endosomes/lysosomes may, for example, digest amphiphilic polymer coatings based on amide bonds [8]. Sée et al. reported the separation of biological molecules from the surface of NPs through peptide bond cleavage by the protease cathepsin L in endosomal compartments [10]. The organic shell or coating of inorganic NPs can also be degraded in lysosomes containing α-glucosidase and other intracellular enzymes. Examples are the dissociation of the carboxydextran shell of ferucarbotran NPs, degradation of methotrexate-modified superparamagnetic NPs, degradation of the polyethylene glycol coating of quantum dots and iron oxide NPs, et cetera [11,12,13]. Degradation of polymeric NPs consisting of poly(-glutamic acid) and l-phenylalanine ethylester by pronase E, protease, cathepsin B, and lipase was also reported, resulting in a decrease in NP size [14]. As pointed out, degradation of the surface chemistry of NPs may have profound effects on their physicochemical properties, involving in particular a loss of colloidal stability and change of the protein corona.

In contrast, enzymatic degradation of NPs can be also used for time-delayed delivery, where molecular cargo is only released after enzymatic degradation of a carrier matrix which encapsulates the cargo [15]. NPs can be formulated in this way to release cargo through enzymes which are only locally present at the target environment. For instance, a polymer/DNA complex for gene therapy was fabricated, in which 4-acetoxybenzyl ester groups were hydrolyzed by esterases, inducing charge-reversal and gene delivery [16]. Due to the high cytosolic esterase activity in HeLa cancer cells but not in fibroblasts, an efficient gene expression in the HeLa cells was reported [16]. Likewise, upregulated matrix metalloproteinase (MMPs) and a lower extracellular pH during the formation of tumors can be used for specific release [17]. Polyvalent nucleic acid/silica NP were designed for intracellular drug delivery based on the opening of the nanopores and the release of the cargo triggered by endonucleases [18]. A better understanding of enzymatic degradation of NP surface chemistries thus also may help for improved degradable delivery vehicles.

As outlined above, there are some reports which describe enzyme-specific cleavage of distinct bonds present on the surface of NPs. Thus, in the present study we wanted to investigate this in more detail for one special case: Inorganic NPs (here, Fe_3_O_4_ NPs) coated with derivatives of the amphiphilic polymer poly-(isobutylene-alt-maleic anhydride)-graft-dodecyl (PMA) [19]. Previously for these NPs, in vitro and in vivo enzymatic degradation of the polymer surface coating has been reported [8]. In the present work, these surface coatings were modified with fluorophores as models for attached ligands, using different coupling chemistries, in order to probe possible enzymatic cleavage of these ligands from the NP surface, as targeting of NPs can depend on the density of targeting ligands attached to the NP surface [20]. Studying potential enzymatic cleavage of ligands from the surface of NPs has relevance for NP-based in vitro and in vivo delivery, or other potential applications.

## 2. Results and Discussion

The synthesis of the Fe_3_O_4_ NPs led to NPs with acceptable size distribution. Much better size distribution may be achieved, but for the present study, the homogeneity of the NP cores was not critical [21]. Transmission electron microscopy (TEM) demonstrated a mean core diameter of d_c_ = (4.4 ± 0.7) nm (see the supporting information, SI §I.1, Figure S1). 

Note that this study does not depend on the choice of Fe_3_O_4_ as the material for the inorganic NP cores. Iron oxide was chosen due to its lower absorption in comparison, for example, to gold NPs of similar size, which facilitates quantification of the number of fluorophores linked per NP by absorption spectroscopy. Thus, no detailed characterization of the chemical structure of the Fe_3_O_4_ cores has been performed here and concerning this, we refer to the original manuscript in which the synthesis has been reported [21]. In fact, in our work we did not confirm Fe_3_O_4_ versus Fe_2_O_3_ stoichiometry, as this is not relevant for this work. For such characterization, we refer to other studies [22,23]. Iron oxide is also a well-studied material, so regarding any particular properties of FeO_x_ NPs, we can refer to the literature [24,25,26,27]. Note also, at that point the Fe_3_O_4_ NPs were dissolved in hexane, and thus no zeta potential measurements in aqueous solution are possible of these iron oxide cores.

**Figure 1:**
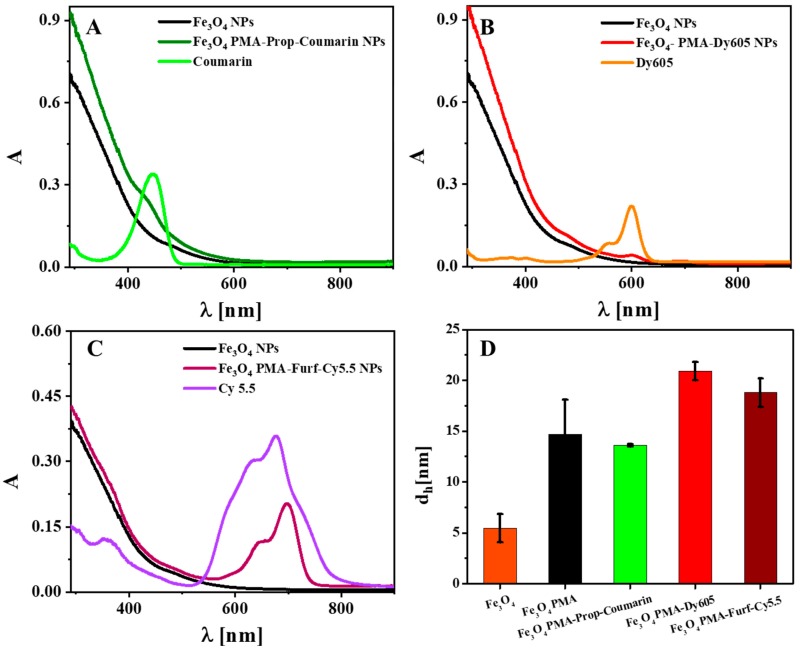
UV-vis absorption spectra A(λ) of (**A**) Fe_3_O_4_ poly-(isobutylene-alt-maleic anhydride)-graft-dodecyl (PMA)-propargylamine (Prop)-Coumarin nanoparticles (NPs), (**B**) Fe_3_O_4_ PMA- Dyomics, #605-02 (Dy605) NPs, (**C**) Fe_3_O_4_ PMA-furfurylamine (Furf)-Cy5.5 NPs, as well as Fe_3_O_4_ PMA and free dyes. (**D**) Hydrodynamic diameter d_h_ of Fe_3_O_4_ NPs as dissolved in chloroform, Fe_3_O_4_ PMA NPs and Fe_3_O_4_ NPs conjugated with three different dyes in water.

Three different PMA polymers were prepared (SI §I.2 and §I.3, Figures S2 and S3), which were modified by dyes using different conjugation chemistries (SI §I.5, Figure S6). To the PMA-coated NPs, amino-modified Dy-605 (Dyomics, #605-02) was bound using EDC chemistry (named as Fe_3_O_4_ PMA-Dy605 NPs), which crosslinks the carboxyl groups present on the PMA (after the anhydride rings have opened in water) with the amino-group of Dy605, leading to an amide bond [28]. The other two conjugation chemistries are Cu(I)-catalyzed azide-alkyne cycloaddition (CuAAC) [29] and the Diels-Alder reaction [30], for the functionalization of propargylamine (Prop)-coated Fe_3_O_4_ NPs with Coumarin and furfurylamine (Furf)-coated Fe_3_O_4_ NPs with Cy5.5, labelled as Fe_3_O_4_ PMA-Prop-Coumarin NPs and Fe_3_O_4_ PMA-Furf-Cy5.5 NPs, respectively, which is known as “click chemistry”. In the case of PMA-Prop-coated NPs, Coumarin 343-azide dye was linked by connecting the azide group of the dye with the alkyne group of Prop, forming 1,4-disubtituted 1,2,3-triazoles. To PMA-Furf-coated NPs, Cy5.5-maleimide dye was attached by reacting the electron-rich diene of Furf with the electron-poor dienophile of Cy5.5-maleimide upon formation of cyclohexene derivative. The NP-dye conjugates were purified, removing unbound polymer and dyes. The hydrodynamic diameters d_h_ and the zeta potentials ζ of the PMA-coated NPs were very similar: Fe_3_O_4_ PMA-Prop NPs: d_h_ = (11.6 ± 0.1) nm, ζ = (−63.4 ± 11.6) mV; Fe_3_O_4_ PMA NPs: d_h_ = (9.7 ± 0.3) nm, ζ = (−57.0 ± 9.41) mV; Fe_3_O_4_ PMA-Furf NPs: d_h_ = (10.4 ± 0.4) nm, ζ = (−64.1 ± 9.63) mV (SI §II.1, Figures S8 and S9). Data demonstrate colloidal stability of the NPs in water without significant agglomeration, with an organic shell thickness of around (d_h_−d_c_)/2 ≈ (10–4.4 nm)/2 = 2.8 nm, in agreement with previous studies [31]. The zeta potential indicated high negative surface charge, though colloidal stability cannot be inferred from this alone. However, the hydrodynamic diameter d_h_ being only slightly bigger than the core diameter d_c_ indicates that in water there cannot be significant agglomeration, as then d_h_ would be increased. Linkage of the dyes increased the hydrodynamic diameters, as it made the NPs less negatively charged (see Figure 1D); Fe_3_O_4_ PMA-Prop-Coumarin: d_h_ = (13.6 ± 0.1) nm, ζ = (−32.8 ± 8.17) mV; Fe_3_O_4_ PMA-Dy605 NPs: d_h_ = (20.9 ± 0.9) nm, ζ = (−38.1 ± 12.1) mV; Fe_3_O_4_ PMA-Furf-Cy5.5: d_h_ = (18.8 ± 1.4) nm, ζ = (−36.9 ± 10.3) mV. The significant increase in hydrodynamic diameter suggests that the bioconjugation reaction induced some small degree of agglomeration of the NPs. UV-vis absorption spectra (Figure 1A–C), fluorescence spectra (SI §II.1, Figure S7), and gel electrophoresis pictures (SI §II.1, Figure S10) clearly demonstrate conjugation of the dyes to the NPs, i.e., the absorption peak of the dyes is visible in the NP-dye conjugates. Quantification of the conjugation based on absorption spectra shows that around 5–8 dye molecules are bound per each NP (SI §II.2, Table S1). Note that this number is prone to error, in particular due to uncertainties in determining the concentration and molar extinction coefficient of Fe_3_O_4_ NPs (SI §I.5, Figure S5), but numbers are in agreement with previous work [32,33].

The dyes were linked to the NPs with three different conjugation chemistries and thus should be prone to enzymatic cleavage by different enzymes. Thus, different enzymes such as fetal bovine serum (FBS), trypsin, cathepsin G (CAT G), lactate dehydrogenase (LDH), aminotransferase (AST), acetylcholinesterase (ACHE), and proteinase K were used in this study to investigate potential enzymatic dye cleavage from the NP surface. For the better understanding of their enzymatic functions, the basic information of these enzymes is listed in the following: (1) Trypsin, an important serine protease, is one of the most important members of the digestive enzymes. It was initially isolated from the pancreatic juice of animals, which has specific functions in food digestion and cellular signal transduction [34,35]. Trypsin is also reported to cleave the C-terminal of arginine and lysine residues, which are converted to ammonia and the corresponding amino acid derivative [36]. (2) CAT G is an endoprotease that belongs to the S1 class of serine proteases, which hydrolyze peptide bonds after leucine, methionine, and phenylalanine residues. It is found in endocytic compartments of various antigen-presenting cells [21]. These proteases are thought to be of high significance in maintaining the delicate balance between tissue protection and destruction for the inflammatory response [37]. (3) LDH is found in almost all animal tissues, in microorganisms, and also in plants. It plays a part in several metabolic pathways, including interconversion of pyruvate to lactate [38]. It has been reported to have five distinct isozymic forms with the same molecular weight but different electrical charges. (4) ACHE, belonging to the primary cholinesterase, is a key molecule in the process of cholinergic transmission. The main physiological task of this enzyme is the fast-hydrolytic destruction of the cationic neurotransmitter acetylcholine to terminate signaling in cholinergic synapses. (5) AST is an important enzyme in the amino acid metabolism that acts by catalyzing the reversible transfer of amino groups between different amino acids [39]. The enzyme exists in soluble (sGOT) and mitochondrial (mGOT) forms and is present in microorganisms, plants, and in all animal and human tissues [40]. The serum AST level is also a common index for liver health. The sample used in the present work is recombinant AST from *E. coli* in a pH 7.5 buffer containing bovine serum albumin (BSA), pyridoxal-phosphate, sucrose, and antibiotics. (6) Proteinase K is a serine protease with 278 amino acids in the polypeptide chain. It cleaves the peptide bond adjacent to the carboxyl group of aliphatic and aromatic amino acids with full enzymatic activity activated by calcium ions [41]. (7) Apart from this, FBS was utilized in our study for probing the degradation of the NPs by various enzymes present in serum. FBS is used in cell cultures to stimulate cellular growth in cell and tissue cultures with components of hormones, vitamins, transport proteins, trace elements, spreading and growth factors, and enzymes [42]. ACHE, LDH, and AST are part of FBS [43].

While the dyes using the three different conjugation chemistries are linked by different types of chemical bonds to the NP surface, all NPs comprise additional amide bonds. For all PMA derivatives, dodecylamine is linked to the polymer backbone via amide bond formation. The conjugation of Fe_3_O_4_ PMA NPs with Dy605 was through amide bonds. In addition, for PMA-Furf and PMA-Prop, the furfurylamine and the propargylamine molecules, respectively, are linked to the polymer backbone by amide bonds. Apart from AST, ACHE, and LDH, all the other enzymes, including FBS, trypsin, CATG, and Proteinase K, are reported to have the ability to cleave amide bonds. 

**Figure 2:**
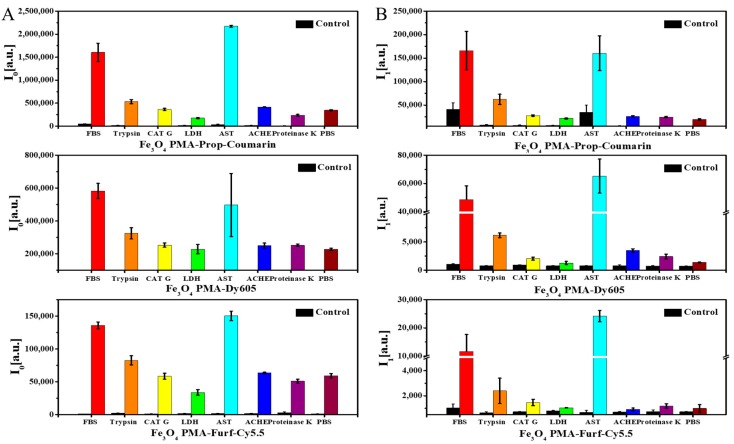
(**A**) Mean emission intensities I_0_ of NP solutions as incubated with enzyme mixes. (**B**) Mean emission intensities I_1_ of small fragments of NPs which, after enzymatic cleavage, were released from the NPs surface and which were collected by ultrafiltration. Data are shown for Fe_3_O_4_ PMA-Prop-Coumarin NPs, Fe_3_O_4_ PMA-Dy605 NPs, and Fe_3_O_4_ PMA-Furf-Cy5.5 NPs “samples” (colored traces) and “controls” (black traces), which represent solutions of enzymes without added NPs.

For measuring enzymatic degradation of the NP coating, the NPs were incubated with enzymes for one day (SI §III.1) before the recording of the fluorescence spectra (SI §III.2 and § III.3, Figures S15 and S16). From the fluorescence spectra, the dye emission intensity I_0_ was determined at λ_max_ (Figure 2A). Solutions of phosphate buffered saline (PBS) without NPs served as controls. As additional controls, all enzymes without the presence of NPs were measured, demonstrating that there was negligible fluorescence of the enzymes. Released dyes and polymer fragments due to enzymatic digestions were then separated by ultrafiltration. Only small molecular fragments, such as the dyes or other parts of the polymer shell, could pass the filter membrane, while the NP cores with the remaining surface coating were retained. Fluorescence intensities of the filtrate are shown in Figure 2B. As reported by others, a quenching of dye due to the close proximity to the iron oxide NPs after conjugation [13] was observed (SI §II.1, Figure S7). Thus, after the enzymatic-driven dye dissociation from the NPs, a fluorescence increase was observed. The residual presence of unidentified enzymes that were present in the commercial forms of BSA in FBS and AST led to an increase of both I_0_ and I_1_ besides the cleavage effect caused by the studied enzymes [44]. 

**Figure 3:**
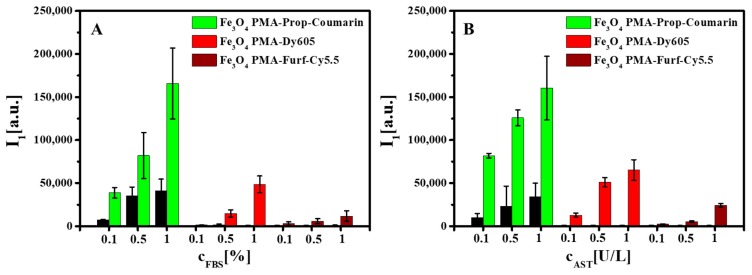
Effect of enzyme concentration on the number of released dye molecules and polymer fragments from the polymer upon enzymatic digestion due to (**A**) FBS and (**B**) AST as presented for Fe_3_O_4_ PMA-Prop-Coumarin NPs, Fe_3_O_4_ PMA-Dy605 NPs, and Fe_3_O_4_ PMA-Furf-Cy5.5 NPs. The black control data refer to control solutions in which the enzymes, but no NPs, were present, after ultrafiltration.

Enzymatic digestion was concentration dependent, i.e., more added enzymes also led to higher dye fluorescence I_1_ in the eluates (Figure 3, SI §III.4, Figures S17–S20). There was, however, also a saturation concentration of enzymes, after which no higher NP digestion could be achieved (SI §III.5, Figure S25). Higher enzyme concentrations in the control samples led to some fluorescence in the eluates. As these solutions did not contain NPs, this fluorescence originated from the enzymes. However, the fluorescence due to the enzymes was much lower than the fluorescence of the enzymatically cleaved dyes. Additional controls, as carried out by inductively coupled plasma-mass spectrometry, demonstrated that only the polymer surface coating, but not the Fe_3_O_4_ NP cores, was subject to enzymatic degradation, i.e., Fe ions were not found in the eluates (see SI §III.5, Figure S24). Thus, the iron oxide cores did not dissolve. We did not verify that enzymes did not cause structural changes in the iron oxide core (for example, Fe_3_O_4_ versus Fe_2_O_3_), as they would not be of relevance for the investigation of the degradation of the NP surface coating. The concentration-dependent data shown in Figure 3 clearly demonstrate enzymatic cleavage of part of the dyes and the polymer shell around the NPs.

**Figure 4:**
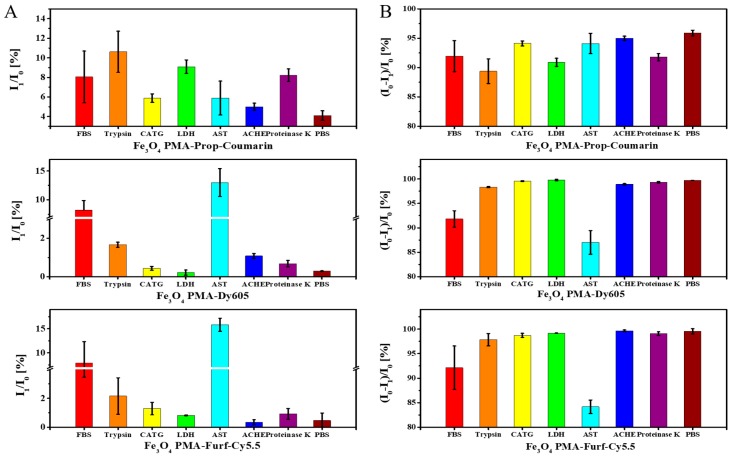
(**A**) I_1_/I_0_ and (**B**) (I_0_-I_1_)/I_0_ of Fe_3_O_4_ PMA-Prop-Coumarin NPs, Fe_3_O_4_ PMA-Dy605 NPs, and Fe_3_O_4_ PMA-Furf-Cy5.5 NPs.

For further quantification, the percentage of fluorescence from dye/polymer fragments cleaved from the NPs (I_1_/I_0_) (Figure 4A) and the percentage of remaining fluorescence of the retained NPs (I_0_ − I_1_)/I_0_) (Figure 4B) are shown. The dependence of the I_1_/I_0_ value on enzyme concentrations is shown in SI §III.4, Figures S21–S23. The data outline shows clear dependence in digestion with the used enzyme. First, no addition of enzymes (i.e., PBS only) did not lead to significant fluorescence in the eluates, indicating that all dyes remained attached to the NPs. FBS and trypsin led to fluorescent eluates for all NP samples. As all dyes are attached either directly via amide bonds, or the linkers of the dyes are attached via amide bonds (as the polymer itself is composed of dodecylamine chains linked to the polymer backbone by amide bonds), all enzymes which may cleave amide bonds (such as trypsin or enzymes present in FBS) led to degradation of the polymer shell. There are, however, some examples for more selective cleavage based on the conjugation chemistries used for linking the dyes. Only in the case of Fe_3_O_4_ PMA-Prop-Coumarin NPs was there enhanced fluorescence in the eluates upon the presence of LDH. On the other hand, AST predominantly acted on Fe_3_O_4_ PMA-Dy605 and Fe_3_O_4_ PMA-Furf-Cy5.5 NPs. Thus, different enzymes may act on dyes linked with different conjugation chemistries to the surface of NPs. 

Understanding the specific interactions between these enzymes and the NPs is arduous, though different reaction mechanisms and enzyme-substrate intermediates are established [45,46]. Besides the reaction specificities, enzymes were also reported to have promiscuity, where enzymes have functions in unexpected or unknown reactions [47]. In this enzyme incubation experiment, based on the result, we assume that LDH can cleave the bond that only exists in backbone of Fe_3_O_4_ PMA-Prop-Coumarin, and AST can catalyze the release of dyes in Fe_3_O_4_ PMA-Dy605 and Fe_3_O_4_ PMA-Furf-Cy5.5 through enzymatic degradation, but the detailed mechanism is not known at this point.

## 3. Materials and Methods

**Figure 5:**
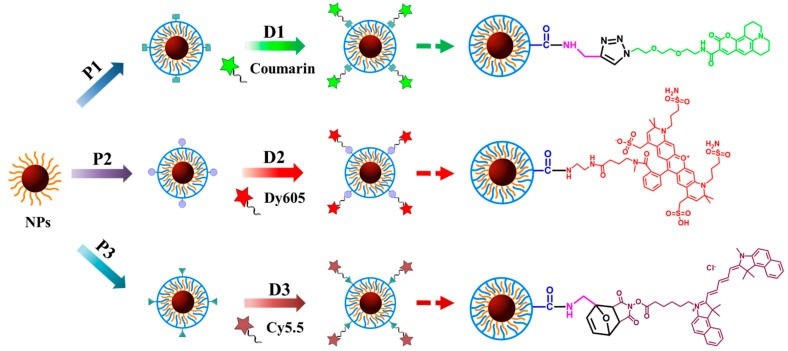
Schematic illustration of the geometry of the used NPs. After the synthesis of hydrophobically capped Fe_3_O_4_ NPs, the NPs were over-coated with three different derivatives of the amphiphilic polymer poly-(isobutylene-alt-maleic anhydride)-graft-dodecyl: PMA-Prop (P1), PMA (P2), and PMA-Furf (P3), leading to dispersion in aqueous phase. Afterward, these NPs were conjugated with different fluorophores: Coumarin 343-azide (D1), Dy605-amine (D2), and Cy5.5-maleimide (D3). On the right, a sketch of the respective coupling chemistries is shown.

Fe_3_O_4_ NPs with core diameters of d_c_ ≈ 4 nm were synthesized with a mixture of 2-hexadecanediol, oleic acid, oleylamine, and phenyl ether, according to protocols from the literature [21]. For a detailed description of all experimental procedures we refer to the Supporting Information (SI §I.1). The NPs were then over-coated according to previously published protocols with different derivatives of PMA, leading to their transfer from the original organic solvent (i.e., hexane) to the aqueous solution (SI §I.2–I.4) [19]. Apart from unmodified PMA (P2 in Figure 5), the following derivatives of PMA were used: Prop and Furf were linked via their amino-groups to PMA before the actual polymer coating procedure, leading to NPs coated with PMA-Prop (P1) and PMA-Furf (P3), respectively.

Unbound excess polymers were removed from the NPs by gel electrophoresis and ultracentrifugation, as has been reported previously (SI §I.4, Figure S4) [19]. The resulting NPs were characterized by dynamic light scattering (DLS) and laser Doppler anemometry (LDA), their absorption and fluorescence spectra were recorded and the amount of fluorophore attached per NP was determined (SI §II.2, Figures S11–S13) [19].

**Figure 6:**
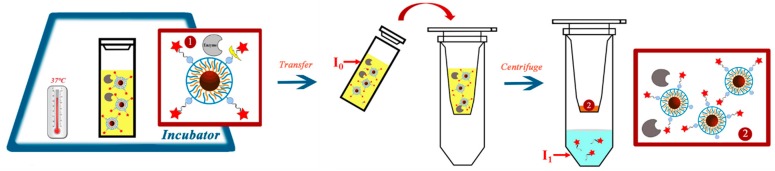
Measurement protocol for recording the fluorescence of the original NP solution (I_0_) and the fluorescence of dyes/polymer fragments released from the NPs upon enzymatic cleavage (I_1_).

The NP solutions were then brought to a defined concentration and volume and were exposed to different enzymes/enzyme-containing solutions in phosphate buffered saline (PBS, pH = 7.4) for 24 h at 37 °C (the final pH values were similar, SI § III.1, Figure S14): FBS (1%), trypsin (0.01%), CAT G (10 U/mL), LDH (10 U/mL), AST (5 U/L), ACHE (10 U/mL), and proteinase K (10 U/mL). PBS was used as enzyme-free control (SI §III.1). After incubation, the fluorescence intensity I_0_ of all samples was determined. The NP/enzyme mixture was then subjected to ultrafiltration with 100 kDa molecular weight cut-off (MWCO) centrifuge filters [8,19]. Here, the PBS buffer, dyes, and small fragments from the polymer shell which had been cleaved by enzymatic degradation from the NPs were eluted, whereas the NPs, due to their larger size, were retained by the filter membrane. The eluent was brought to the initial volume of the original solution and then the fluorescence I_1_ was recorded. The measurement protocol is summarized in Figure 6.

## 4. Conclusions

Polymer shells around inorganic NP cores with attached dyes may be subject to enzymatic degradation. Degradation will depend on the types of enzymes the NPs encounter, as well as the chemistry of the polymer and the conjugation chemistry with which dyes are linked to the polymer shell. Understanding the detailed mechanism of such enzyme specific degradation remains a great challenge to consider in the future. Concerning the use of the NPs in biological scenarios, one should always take into account the enzymes which the NP may encounter during its lifecycle, as enzymatic digestion of the NP surface coating may significantly vary the functional and physicochemical properties of the NPs. This enzymatic degradation must be considered especially when analyzing the performance of biomolecules (i.e., antibodies, antifouling agents, drugs, carbohydrates, etc.) bound to the NP´s surface using any of these bioconjugation strategies. 

## Supplementary Materials

Supplementary materials can be found at https://www.mdpi.com/1422-0067/20/4/935/s1.
