# Supplementary material for: Investigating Possible Enzymatic Degradation on Polymer Shells around Inorganic Nanoparticles
Source: Int J Mol Sci. 2019 Feb 21;20(4):935. doi: 10.3390/ijms20040935 (PMC6412445; doi:10.3390/ijms20040935)
Supplement: Supplementary file 1 [file ijms-20-00935-s001.pdf]

Supporting information

Investigating possible enzymatic degradation on polymer shells around inorganic nanoparticles

Lin Zhu[1], Beatriz Pelaz[2], Indranath Chakraborty[1], Wolfgang J. Parak[1,3*]

[2]Faculty of Physics, Center for Hybrid Nanostructures (CHyN), Universität Hamburg, Hamburg, Germany

[2]University of Santiago de Compostela, Santiago de Compostela, Spain

[3]CIC Biomagune, San Sebastian, Spain

[*]corresponding author: wolfgang.parak@uni-hamburg.de

I. Materials and methods
I.1 Synthesis of hydrophobic Fe_3O_4 nanoparticles (NPs)
I.2 Synthesis of dodecylamine modified poly(isobutylene-alt-maleic anhydride) (PMA)
I.3 Modification of PMA with furfurylamine and propargylamine
I.4 Polymer coating and purification of Fe_3O_4 NPs
I.5 Conjugation of Fe_3O_4 NPs with different dyes

II. Characterization and quantification
II.1 Characterization of dye-conjugated Fe_3O_4 NPs
II.2 Quantification of dye conjugation

III. Enzyme-induced degradation of the polymer shell of dye conjugated Fe_3O_4 NPs
III.1 NPs incubation with enzymes
III.2 Fluorescence spectra of NPs incubated with enzyme (I_0)
III.3 Fluorescence spectra of released dye in the eluate (I_1)
III.4 Dependence on enzyme concentration
III.5 Control experiments

IV. References

I Materials and methods

I.1 Synthesis of hydrophobic Fe_3O_4 nanoparticles (NPs)

To synthesize Fe_3O_4 NPs cores with diameter of ~4 nm, a thermal decomposition reaction was performed following the methodology reported by Sun *et al* [1]. In brief, the synthesis was carried out in an oxygen- and water-free glovebox. Under a blanket of nitrogen, $Fe(acac)_3$ (2 mmol, Sigma Aldrich, #517003), 1, 2-hexadecanediol (10 mmol, Sigma Aldrich, #213748), oleic acid (6 mmol, Sigma Aldrich, #O1008), oleylamine (6 mmol, Sigma Aldrich, #O7805), and phenyl ether (20 mL, Sigma Aldrich, #108014) were mixed and stirred at 200 °C for 30 min. After that, the mixture was heated up to 265 °C for another 30 min. In this reaction, $Fe(acac)_3$ is used as metal precursor, which upon thermal decomposition is reduced by 1,2-hexadecanediol into Fe_3O_4 NPs. Finally, the black-brown mixture was cooled down to room temperature and taken out from the glovebox. For the purification of the Fe_3O_4 NPs, the product was separated into two 50 mL centrifuge tubes and then 25 mL of ethanol was added to each tube. Centrifugation at 6000 rpm for 10 min was applied and supernatants were discarded. The black precipitate was dissolved in 20 mL hexane with oleic acid (0.05 mL) and oleylamine (0.05 mL) in each tube. The centrifugation process was performed again and the Fe_3O_4 NPs were finally re-dispersed in hexane. Note that no analysis to confirm Fe_3O_4 structure was carried out, as the precise nature of the iron oxide core is irrelevant for this work. The corresponding transmission electron microscopy (TEM) images of the NPs dried on a TEM grid are presented in Figure S1 and confirm good monodispersity of the NPs with an average core diameter of d_c = (4.4 ± 0.7) nm.

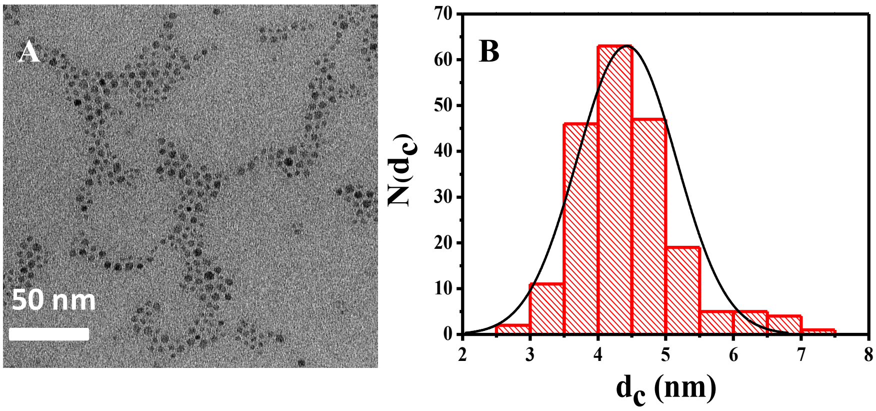

Figure S1. *(A) TEM image of hydrophobic Fe_3O_4 NPs, and (B) the size distribution diagram $N(d_c)$ of the core diameter d_c as obtained from Image J analysis. The average diameter, d_c of the Fe_3O_4 NP core was determined to be (4.42 ± 0.73) nm.*

I.2 Synthesis of dodecylamine modified poly(isobutylene-alt-maleic anhydride) (PMA)

Amphiphilic polymers have been widely used for the over-coating of NPs with the purpose of transferring hydrophobic NPs from organic solution to aqueous solution [2-4]. There are a large variety of amphiphilic polymers that can be used to coat NPs. In this work, dodecylamine modified poly(isobutylene-alt-maleic anhydride) (PMA) was selected based on previous work, which consists of dodecylamine hydrophobic side chains for interfacing the NP surface and a hydrophilic backbone of poly(isobutylene-*alt*-maleic anhydride) [2]. The hydrophobic dodecylamine side chains bearing amino groups were linked to 75% of the anhydride rings of the hydrophilic backbone in a one-pot reaction, leaving 25% intact anhydride rings [5]. During the polymer coating procedure, the hydrophobic side chains intercalate the hydrophobic surface capping of the NPs, and the leftover 25% anhydride rings of the hydrophilic backbone open up in basic condition yielding negatively charged carboxyl groups, which make the NPs soluble in aqueous medium. The electrostatic repulsion of the individual NPs due to the negatively charged amphiphilic polymer leads to a stable NP dispersion in water.

For the synthesis of PMA (a scheme is given in Figure S2), 2.70 g (15 mmol) dodecylamine (Sigma-Aldrich, #325163) were dissolved in 100 mL anhydrous tetrahydrofuran (THF, Sigma-Aldrich, #401757) in a 250 mL round bottom flask. Then, 3.084 g poly(isobutylene-*alt*-maleic anhydride) (average molecular weight of monomer unit M_w ~6,000 g/mol, Sigma-Aldrich, #531278) was added to this flask. After sonication of this mixture for 20 s, it was refluxed at 55-60 °C under constant stirring (800 rpm) for 3 h. Then, the solution was concentrated to around 30 mL by evaporation using a rotavapor (Heidolph, Laborota 4003 control) and then refluxed overnight for the reaction of the PMA backbone with dodecylamine. Finally, the solvent was completely evaporated and the product was dissolved in 40 mL anhydrous chloroform to get the final concentration of c_p = 0.5 M of polymer monomer units [5].

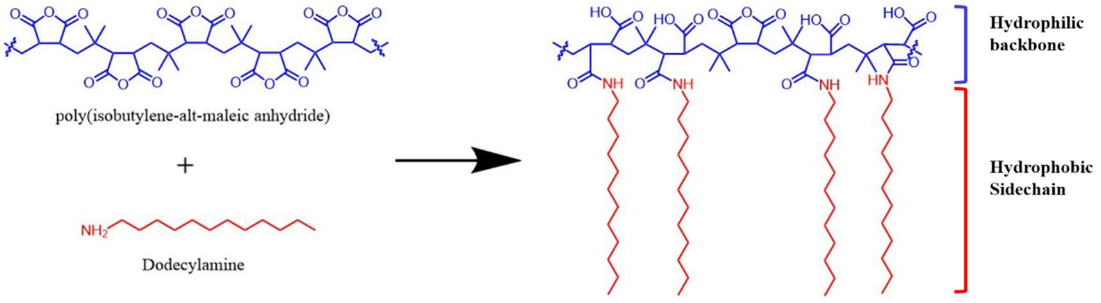

Figure S2. *Reaction scheme of the PMA synthesis. The hydrophilic backbone and hydrophobic dodecylamine side chains are presented in blue and red color, respectively.*

I.3 Modification of PMA with furfurylamine and propargylamine

Similar to the linkage of dodecylamine to the hydrophilic backbone of PMA, molecules with an amino group can be linked *via* amide bonds to the maleic anhydride rings [6, 7]. This method was used to add further molecular anchors, here furfurylamine and propargylamine, to the polymer, which later-on were used to attach different fluorophores. After the synthesis of PMA, 25% intact anhydride rings of the amphiphilic polymer can still be utilized to link furfurylamine or propargylamine. Here, to link these molecules to PMA, 2% of the total anhydride rings of the amphiphilic polymer were firstly modified by the reaction of the maleic anhydride rings with the amino groups of furfurylamine or propargylamine molecules, which is described schematically in Figure S3. In details, 10 mL of c_p = 0.5 M amphiphilic polymer (concentration referring to the monomer units) in chloroform was mixed with a solution of 0.1 mmol furfurylamine (Sigma-Aldrich, F20009) or 0.1 mmol propargylamine (Sigma-Aldrich, P50900) in a round flask and the reaction mixture was refluxed at 55-60 °C overnight. Afterwards, the solvent mixture was evaporated with a rotavapor and the modified polymer was redissolved in 20 mL anhydrous chloroform to obtain a final polymer monomer concentration of c_p = 0.25 M. As it has been mentioned, 75% of the anhydride rings were reacted with dodecylamine as described, and thus after linkage of 2% of the anhydride rings with furfurylamine or propargylamine, *i.e.* in total 23% of the anhydride rings remained unreacted. The amphiphilic polymer linked with furfurylamine and propargylamine is in the following termed as PMA-Furf and PMA-Prop, respectively. Note that 100% reaction efficiency was assumed, though actual reaction efficiencies may be less [8].

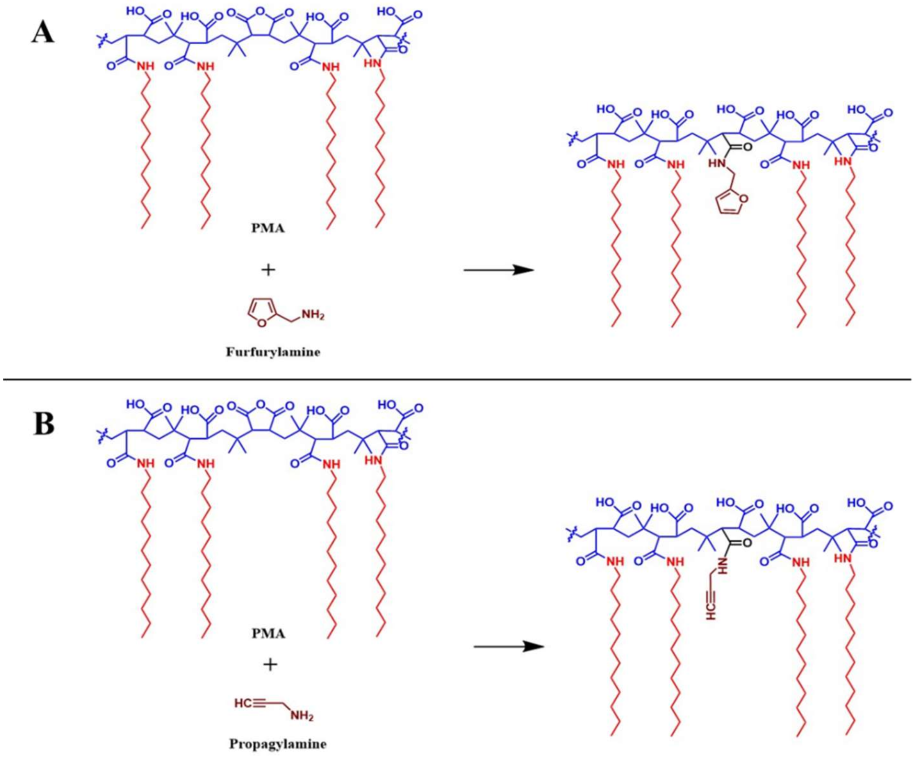

Figure S3. *Schemes of PMA surface functionalization with (A) furfurylamine and (B) propagylamine. The red and blue color demonstrates the hydrophilic and hydrophobic parts, respectively.*

I.4 Polymer coating and purification of Fe_3O_4 NPs

The polymer coating was carried out as reported before [2, 3]. The amount of polymer solution V_P added to the NPs was determined as:

$$V_p = \frac{R_{P/Area} \times \pi \times {d_{eff}}^2 \times c_{NP} \times V_{NP}}{c_p} \quad .$$

Here c_{NP} (cf. Chapter I.5) and V_{NP} are the concentration and the volume of the NP solution, respectively. In this order, c_p and V_p are the monomer concentration and the volume of the amphiphilic polymer solution. d_{eff} is the effective diameter of NPs including the diameter of inorganic core and twice the thickness hydrophobic surfactant shell: $d_{eff} = d_c + 2 \cdot l_{ligand}$. Here $d_c = 4.4$ nm as determined by TEM and $l_{ligand} = 1$ nm were used. $R_{P/Area}$ is the ratio of polymer units *per* nm^2 of effective NP surface. For the Fe_3O_4 NPs in the present study, the value $R_{P/Area} = 100\ nm^{-2}$ was chosen [5].

Polymer coating with the three different polymers (PMA, PMA-Furf, and PMA-Prop) was carried out in chloroform [5]. First, the hydrophobic Fe_3O_4 NPs in chloroform were

mixed with PMA, PMA-Furf, and PMA-Prop separately in three different flasks. Each solution was stirred manually for 5 min and then the solvent was completely evaporated in a rotary evaporator under heating to 40 °C in order to force the polymer to wrap around the NPs. To obtain a homogeneous coating, a few mL of anhydrous chloroform was added to the flask to reconstitute the solid film and again the solvent was removed under reduced pressure. After that, alkaline sodium borate buffer (SBB 12, 50 mM, pH 12 adjusted with NaOH) was added and the mixture was vigorously stirred until the solution turned clear. In this way, all the NPs were transferred into SBB 12 solution [5].

After the polymer coating, there was free PMA in the NP solution [9]. Thus, a cleaning process was carried out to warrant for the purity of Fe_3O_4 NPs. Firstly, the NP samples in SBB 12 were cleaned by using centrifugal filters (15 mL, Amicon Ultra, 100 kDa) at 4000 rpm for 15 min [5]. After concentrating the samples, loading buffer (20% of the sample by volume) for gel electrophoresis was added to the samples. The loading buffer was prepared by mixing 35 mL 0.5× tris-borate-EDTA buffer (TBE, Sigma-Aldrich, #T3913), 25 mL glycerol (Sigma-Aldrich, # G8773) and 130 mg Orange G (Sigma-Aldrich, # 861286). The mixture then was loaded on a 2% agarose gel in a Tris-Borate-EDTA buffer (TBE 0.5x, Sigma-Aldrich, # T3913) for running of gel electrophoresis at 110 V for 1 h [5]. Figure S4 represents the corresponding gel pictures of Fe_3O_4 PMA NPs, Fe_3O_4 PMA-Prop NPs, and Fe_3O_4 PMA-Furf NPs. Due to the negative charge of the PMA, the NPs showed good electrophoretic mobility. The narrow band corresponding to the NPs on the gel confirmed the good monodispersity of the NPs. Some free polymers can still remain in the solution after gel electrophoresis [5]. Thus, ultracentrifugation was also carried out for three times to make sure of the removal of unbound polymers (54000 revolutions *per* minute (rpm), 1 h).

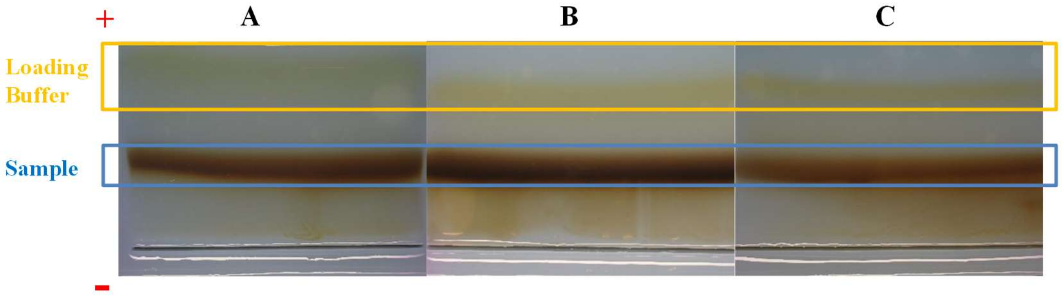

Figure S4. *Gel photographs of (A) Fe_3O_4 PMA NPs, (B) Fe_3O_4 PMA-Furf NPs, and (C) Fe_3O_4 PMA-Prop NPs. Images were taken by a BioRad Gel Doc imaging device. The samples were run at 110 V for 1 h. The NP samples were first mixed with loading buffer. After the application of voltage, the negatively charged NPs run through the pre-made 2% agarose gel towards the positive pole.*

I.5 Conjugation of Fe_3O_4 NPs with different dyes

To conjugate the dyes to the Fe_3O_4 NPs, different chemistry including "click chemistry" was carried out and analyzed. Click chemistry has been used to synthesize multiple biomaterials [10]. It takes place usually in room atmosphere and is insensitive to water and oxygen [11]. In our experiments the Cu(I)-catalyzed azide-alkyne cycloaddition (CuAAC) reactionand the Diels-Alder reaction were performed for the linkage of Fe_3O_4 PMA-Prop NPs with Coumarin and Fe_3O_4 PMA-Furf NPs with Cy5.5, respectively [12, 13]. The original "click chemistry", referred to broadly as CuAAC, was first introduced to by Sharpless in 2001 [11]. The CuAAC reaction has broad applications in medicinal chemistry for the linkage of peptides, nucleotides, small molecules, supramolecular structures, polymers, *etc* [14]. The Diels-Alder reaction is also one of the most common "click chemistry" strategies, discovered by Otto Diels and Kurt Alder between a conjugated diene and a substituted alkene [15]. Beside these strategies, the Fe_3O_4 PMA NPs were furthermore conjugated with amine-modified Dy-605 *via* 1-Ethyl-3-(3-dimethylaminopropyl)carbodiimide (EDC) chemistry, in which EDC is used as a carboxyl activating agent to couple amines [16, 17].

Before the reaction, the concentration c_{NP} of the Fe_3O_4 PMA NPs was calculated by Beer-Lambert's law using

$$c_{NP} = \frac{A}{\varepsilon_{NP} \times l}$$

Here A is the absorbance of NPs at 450 nm and ε is the extinction coefficient of the NPs

at 450 nm, l is the path length of the cuvette, which is 1 cm in our experiments [3].

The calculation of the molar extinction coefficient been published previously [7, 18]. In the following the numbers for one measurement are given as example. The mass concentration of Fe in one sample was measured by inductive coupled plasma mass spectrometry (ICP-MS) as C_{Fe} = 0.0889 mg/mL. From the chemical formula of Fe_3O_4, the atom ratio of iron to oxygen is 3/4. Therefore, the content of the oxygen is C_O = $4/3 \times C_{Fe} \times (M_O \times M_{Fe})$ = 0.03378 mg/mL, using the molar mass of oxygen, M_O = 15.9 g/mol, and of iron, M_{Fe} = 55.8 g/mol. The total mass concentration of the investigated sample thus was $C_{NP} = C_{Fe} + C_O$ = 0.1227 mg/mL.

To calculate the molar extinction coefficient, the molecular weight M_{NP} of the NPs has also to be estimated. By the assuming that Fe_3O_4 NPs are spheres of 4.4 nm core diameter (d_c = 4.4 nm), the volume of one NP core is $V_{Fe3O4} = (4/3) \times \pi \times (d_{core}/2)^3 = 4.46 \times 10^{-20}$ cm^3. The density of Fe_3O_4 is ρ_{Fe3O4} = 5.18 g/cm^3. Then, the mass of each Fe_3O_4 NP core is $m_{NP} = \rho_{Fe3O4} \times V_{Fe3O4} = 2.31 \times 10^{-19}$ g. From the mass of one NP, the molecular weight M_{NP} can be determined as $M_{NP} = m_{NP} \times N_A = 1.39 \times 10^5$ g/mol, with the Avogadro number $N_A = 6.022 \times 10^{23}$ mol^{-1}. The molecular concentration of the measured sample was thus $c_{NP} = C_{NP}/M_{NP}$ = 0.883 μM.

Finally, the UV-vis absorption of different dilutions of the NP solutions was collected, and the UV-vis absorption at 450 nm versus the NP concentration was fitted with a linear function in Figure S5. The number for $\varepsilon_{Fe3O4}(450)$ for Fe_3O_4 at the wavelength of 450 nm is about 1.17×10^6 M^{-1}cm^{-1}.

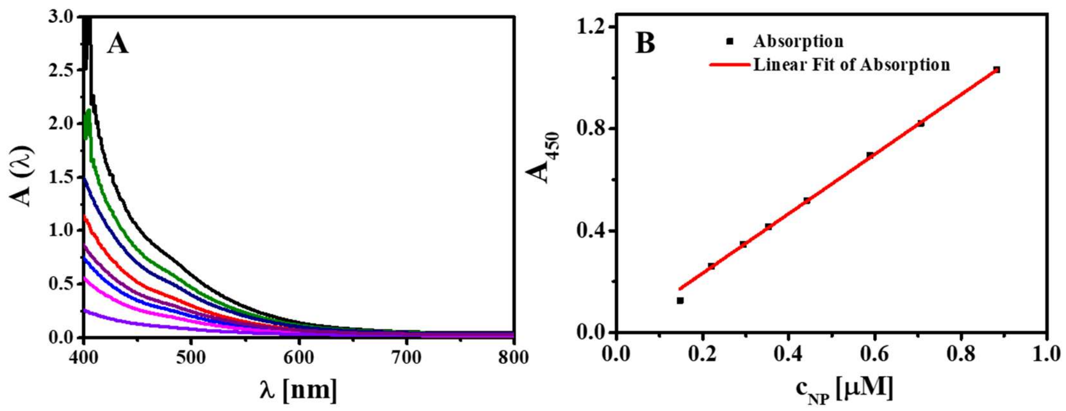

Figure S5. *(A) UV-vis absorption spectra A(λ) of hydrophilic Fe_3O_4 NPs after polymer coating at different dilutions. (B) The absorption at 450 nm versus the concentration of Fe_3O_4 NPs was recorded separately. The cure was fitted linearly with the experimental data to calculate the molar extinctions coefficient (ε).*

After the concentration determination of Fe_3O_4 NPs the dye conjugation was performed. The reactions are individually presented in Figure S6: CuAAC reaction (Figure S6 A), Diels-Alder reaction (Figure S6 B), and EDC chemistry (Figure S6 C). The CuAAC reaction is a 1,3-dipolar cycloaddition for generating 1,4-disubtituted 1,2,3-triazoles between terminal alkynes and azides using Cu(I) salts as a catalyst [19]. In our experiment, the Fe_3O_4 PMA-Prop NPs containing terminal alkyne groups were reacted with azide-modified Coumarin. Ascorbic acid was added to the copper sulfate to reduce Cu(II) to Cu(I), as Cu(I) is the reaction catalyst. The Diels-Alder reaction involved a cycloaddition between Fe_3O_4 PMA-Furf NPs with an electron-rich diene and Cy5.5 with an electron-poor dienophile, and the final product they formed is a cyclohexene derivative. In the third reaction, Fe_3O_4 PMA NPs containing carboxyl groups were firstly activated by EDC, which is a water-soluble carbodiimide. Then the activated carboxyl group was conjugated with the amine group in Dy605 to yield an amide group. The reaction details are explained in the following.

The concentration of the stock solution of Fe_3O_4 PMA-Prop NPs was 20.5 µM, a ratio of 25 molecules of coumarin 343 azide dye (Lumiprobe, #A1630, Excitation Wavelength: 437 nm) *per* NP was chosen for functionalization. 117 µL (2.4 nmol) Fe_3O_4 PMA-Prop NPs were taken from the initial batch and mixed with 26.4 µg (60 nmol dissolved in water) coumarin. Cu(I) as the catalyzing agent was prepared by mixing 16 mg copper sulfate (Sigma-Aldrich, #451657) and 88 mg of ascorbic acid (Sigma-Aldrich, #A92902) for a few minutes in 10 mL of Milli-Q water. Then 2.5 µL of this solution were added in the reaction as reported previously [19]. After dilution with water, the reaction was in a final volume of 1 mL. The final concentration of Fe_3O_4 PMA-Prop NPs was 0.15 µM. This mixture was left under agitation overnight.

For Fe_3O_4 PMA NPs at the concentration of 17.6 µM, 136 µL of Fe_3O_4 PMA NPs (2.4 nmol) were reacted with Dy-605 (Dynomics, #605-02) using EDC (Sigma-Aldrich, #E6383) at a ratio of 4000 molecules of EDC *per* molecules of NPs (Figure S6 C). Thus, 2.3 mg of EDC was dissolved in 230 µL SBB 9 and then mixed with the Fe_3O_4 PMA NPs for a few minutes in order to activate the carboxylic groups. Afterwards, 68 µg Dy-605 (60 nmol) amino modified dye was mixed in the solution. The ratio of dye to NP was about 25. The final reaction concentration of Fe_3O_4 PMA NPs was 3 µM after diluting the solution with SBB 9 to a final volume of 1 mL. The reaction lasted for 2 hours before cleaning.

Fe_3O_4 PMA-Furf NPs (36.4 µM) were conjugated with the Cy-5.5 maleimide dye (Lumiprobe, #17080, excitation wavelength: 673 nm) in 2-(N-morpholino)ethanesulfonic acid (MES, Sigma-Aldrich, #M3671, 100 mM, pH 6) (Figure S6 B). 61.8 µL (2.25 nmol) Fe_3O_4 PMA-Furf NPs was mixed with of 32 µg (45 nmol) Cy5.5 and brought to a final volume of 4 mL in MES. In this case, the ratio of dye *per* NPs was reduced to 20. When higher concentration of dye *per* NPs was used in this experiment, NP aggregates occured in the solution. In addition, the reaction time

was cut down to 45 min, probing the efficiency of this reaction. The mixture was taken for cleaning immediately after reaction.

After all the reactions, the unreacted dye molecules were washed out. This is a critical step as non-covalently bond dyes would falsify the degradation results. Firstly, each sample was cleaned by membrane dialysis (10 kDa, Spectrum, #G235055) in 10 mM NaOH solution for 4 h, where unbound dye could diffuse out of the dialysis bath, whereas the NPs were retained [5]. Then, the dialysis bath was changed and the dialysis step was repeated 2 times. Next, the sample was collected and washed with centrifuge filters (5 mL, 100 kDa molecular weight cut-off (MWCO)) at 4000 rpm for 10 min [5]. This step was repeated until there was basically no longer free dye in the eluent.

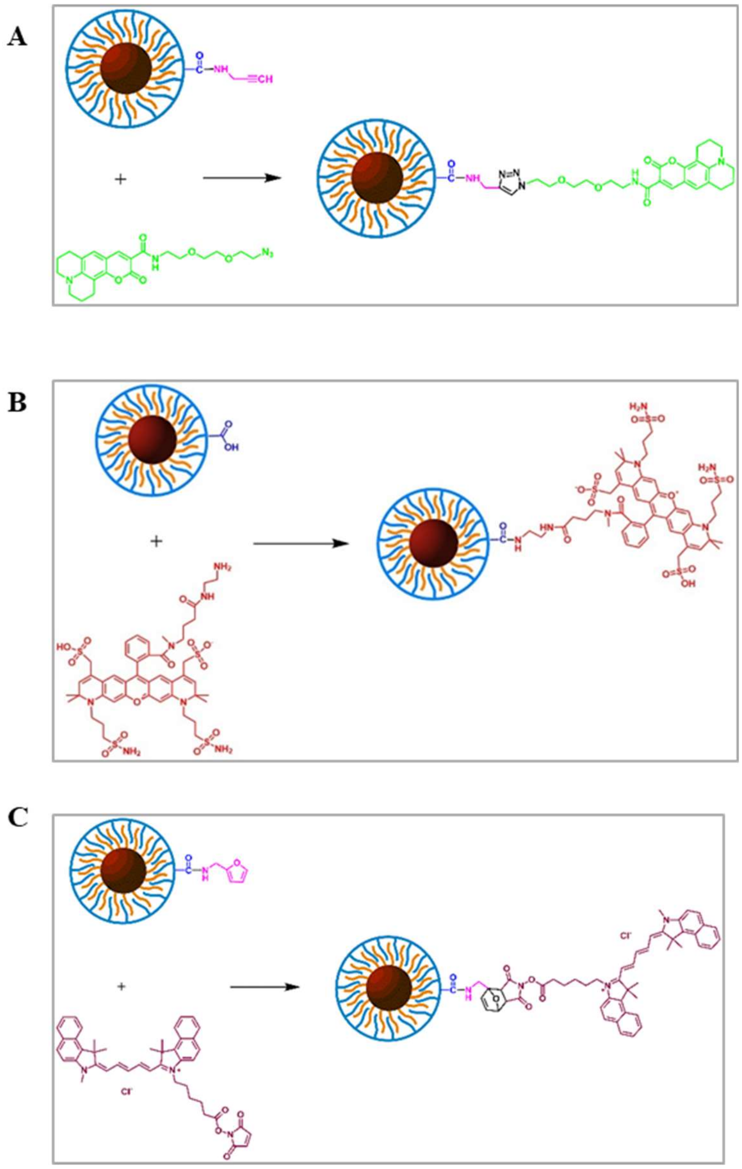

Figure S6. *Structure of conjugation of (A) Fe_3O_4 PMA-Prop-Coumarin, (B) Fe_3O_4 PMA-Dy605 NPs, and (C) Fe_3O_4 PMA-Furf-Cy5.5 NPs.*

II Characterization and quantification

II.1 Characterization of dye-conjugated Fe_3O_4 NPs

All NPs were characterized using different spectroscopic and microscopic techniques. Nuclear magnetic resonance (NMR) and infrared (IR) characterizations were attempted. However, no meaningful information was obtained for the here described dye conjugated NPs, since each polymer has multiple functional groups, which makes quantification of dye-conjugation complexes complicated. Instead, emphasis was given to fluorescence and gel electrophoresis data for the confirmation of the polymer coating and the dye conjugation. This gave clear results, as the Fe_3O_4 NPs without attached dye are not fluorescent.

Dye conjugation of Fe_3O_4 NPs was confirmed from their absorbance, fluorescence, dynamic light scattering (DLS), zeta potential, and gel electrophoresis analysis data. In each case, the absorbance spectra of conjugated NPs showed the presence of the characteristic dye features (*cf.* Figure 1 of the main manuscript). Since NPs without dyes are not fluorescent, fluorescence spectroscopy is a good way to confirm the dye conjugation. Similar to absorption spectra, the spectral fluorescence features of the dyes were seen in each of the spectra recorded of dye conjugated NPs (Figure S7). For the case of Cy5.5 conjugation, a substantial shift of the fluorescence peak (~20 nm) was seen, which might be due to some structural re-arrangement of the dye upon conjugation [20]. The decrease of luminescent intensity from free dyes to NP-dye conjugates can be result of re-absorbance of the NPs. The fluorescence spectral features of dyes were seen in each dye conjugated NPs. The quenching effect of dyes attached on NPs was reported by Jang *et al* [21].

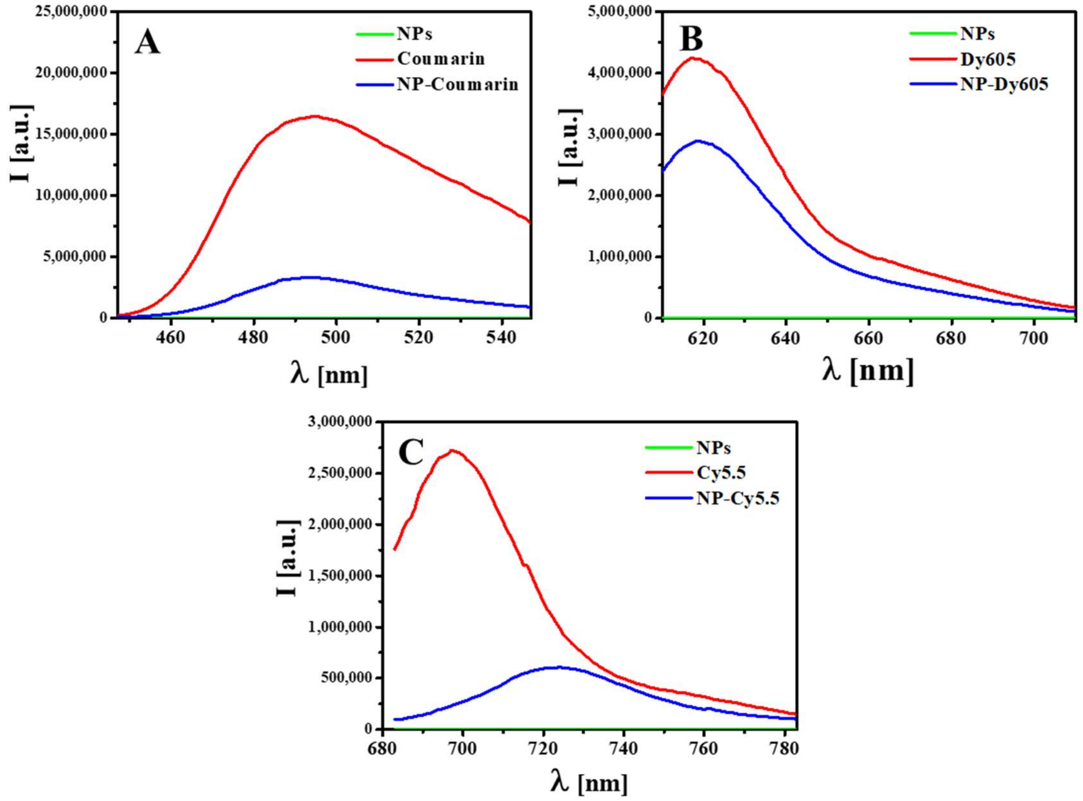

Figure S7. *Comparative fluorescence spectra of Fe_3O_4 NPs (green trace), free dye (red trace), and Fe_3O_4 NPs conjugated with dye (blue trace) of (A) Fe_3O_4 PMA-Prop-Coumarin NPs, (B) Fe_3O_4 PMA-Dy605 NPs, and (C) Fe_3O_4PMA-Furf-Cy5.5 NPs.*

After the removal of free dye, the hydrodynamic diameter distribution and the zeta potential of the Fe_3O_4 NPs before and after dye conjugation were determined *via* DLS by a Zetasizer Nano ZS Malvern Instruments in Figures S8 and S9. All the values are calculated as average of at least three measurements with corresponding standard deviation. The hydrodynamic diameter d_h of Fe_3O_4 coated with PMA-Prop, PMA, PMA-Furf was found to be 9-12 nm. After conjugation with coumarin, Dy605 and Cy5.5, there were increases in their size at different level, with the final d_h value between 13 to 21 nm. The increase of the size is mainly caused by the dye attachment, but also will involve some slight agglomeration effects. After dye conjugation, also in the zeta potential measured an obvious change was observed, which varied from approximately -60 mV to -35 mV. The decrease of zeta potential may not only be result of the dye attachment but may be due to reduction in the density of the polymer coating during the intensive cleaning process which was carried out in order to remove free dye.

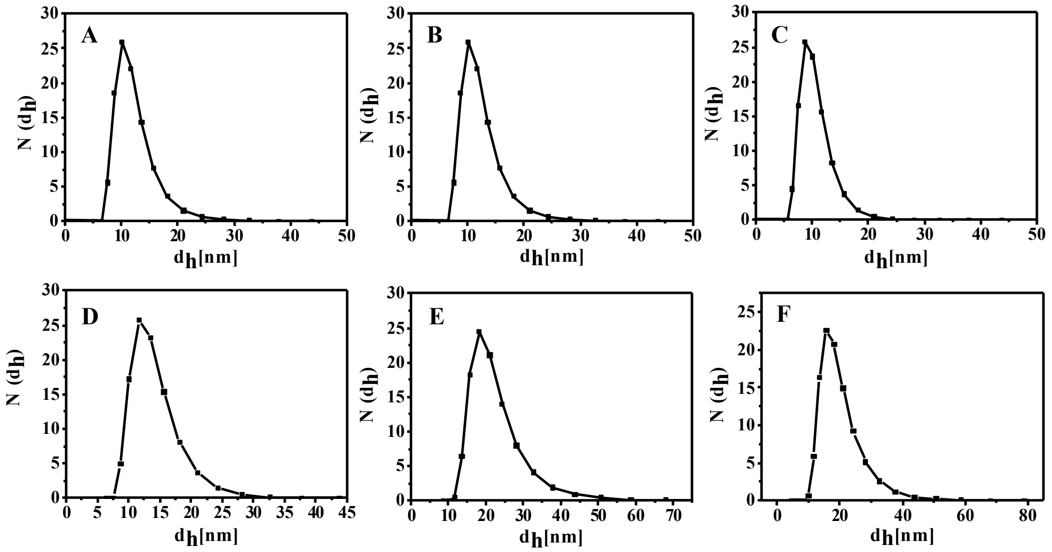

Figure S8. *Number distribution of the hydrodynamic diameter d_h (bottom row) of Fe_3O_4 NPs before and after dye conjugation in aqueous solution. (A) Fe_3O_4 PMA-Prop d_h = 11.6 ± 0.1 nm. (B) Fe_3O_4 PMA d_h = 9.7 ± 0.3 nm. (C) Fe_3O_4 PMA-Furf d_h = 10.4 ± 0.4 nm. (D) Fe_3O_4 PMA-Prop-coumarin d_h = 13.6 ± 0.1 nm. (E) Fe_3O_4 PMA-Dy605 d_h = 20.9 ± 0.9 nm. (F) Fe_3O_4 PMA-Furf-Cy5.5 d_h = 18.8 ± 1.4 nm.*

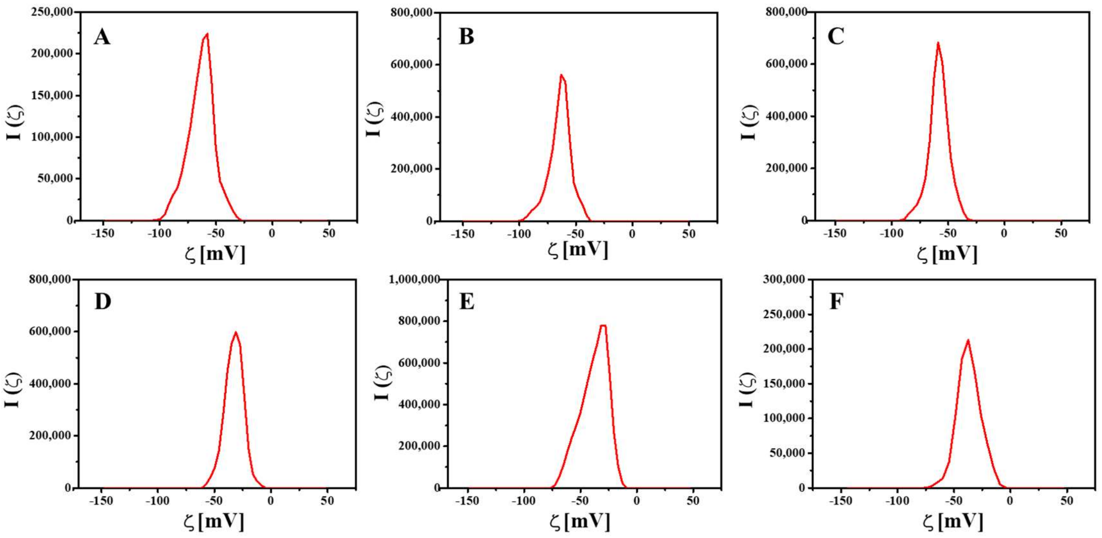

Figure S9. *Distribution of zeta potential ζ of Fe_3O_4 NPs before and after dye conjugation as measured in aqueous solution. (A) Fe_3O_4 PMA-Prop ζ = -63.4 ± 11.6 mV. (B) Fe_3O_4 PMA ζ = -57.0 ± 9.41 mV. (C) Fe_3O_4 PMA-Furf ζ = -64.1 ± 9.63 mV. (D) Fe_3O_4 PMA-Prop-coumarin ζ = -32.8 ± 8.17 mV. (E) Fe_3O_4 PMA-Dy605 ζ = -38.1 ± 12.1 mV. (F) Fe_3O_4 PMA-Furf-Cy5.5 ζ = -36.9 ± 10.3 mV.*

After the purification of unbound excess dye, gel electrophoresis was carried out to further confirm the conjugation of the NPs with dye. Dyes used in this work are nearly neutral and therefore significant migration was not seen, whereas conjugated NPs

moved towards the positive pole due to their negative net charge originating from the polymer shell, see Figure S10.

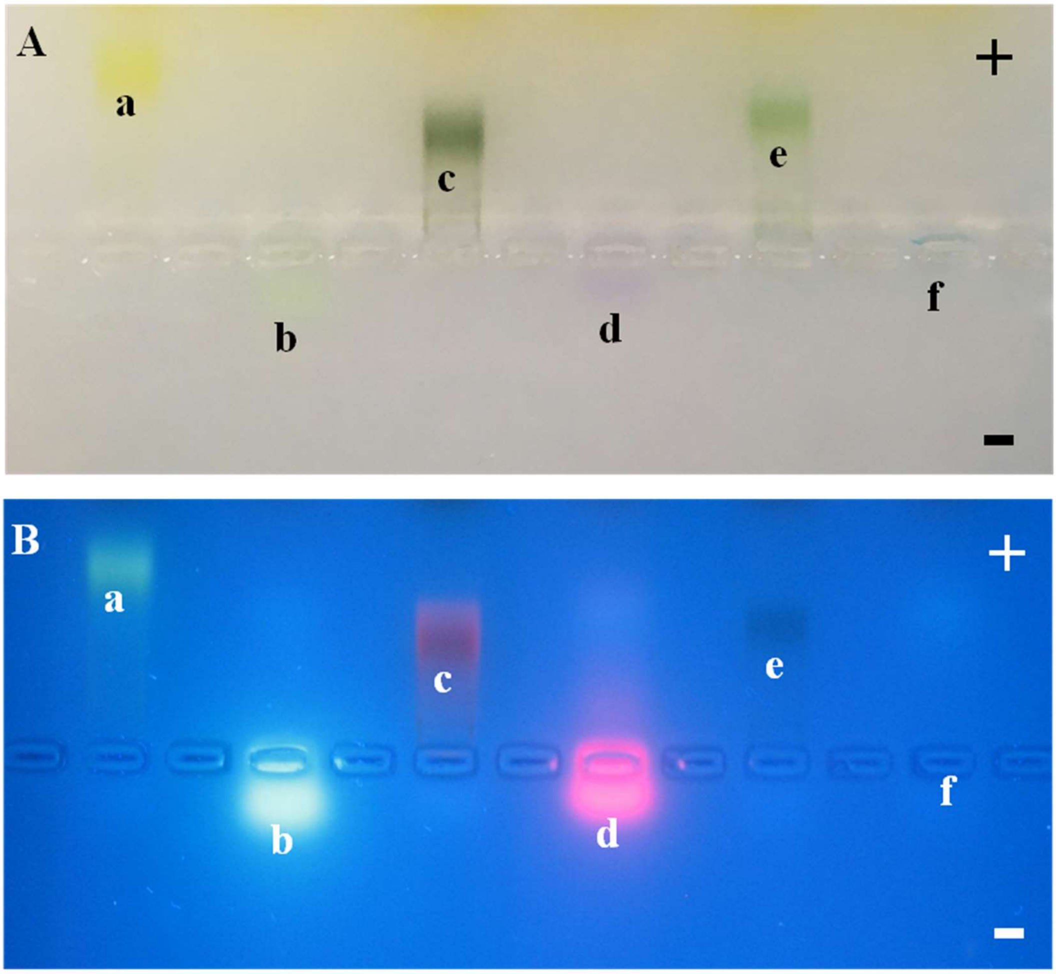

Figure S10. *Photographs 2% agarose gels after running for 0.5 h at 110 V recorded under (A) visible and (B) UV light. a) Fe_3O_4 PMA-Prop-coumarin NPs, b) coumarin, c) Fe_3O_4 PMA-Dy605 NPs, d) Dy605, e) Fe_3O_4 PMA-Furf-Cy5.5 NPs, f) Cy5.5.*

II.2 Quantification of dye conjugation

In each case, the ratio of dye *per* NP was calculated using Beer-Lambert's law and the corresponding UV-vis absorption mentioned in §I.5. Absorbance was recorded at wavelengths with minimized overlap of NP and dye absorption. Absorbance at 450 nm A_{450} was used for determining the concentration of Fe_3O_4 PMA-Dy605 NPs and Fe_3O_4 PMA-Furf-Cy5.5 NPs, since the corresponding free dyes do not have significant absorbance at this wavelength (Table S1). For Fe_3O_4 PMA-Prop-coumarin NPs, absorbance A_{500} for determining the NP concentration was collected at 500 nm instead of 450 nm, as coumarin has its absorbance peak near 437 nm and has hardly any absorbance at 500 nm. The concentration ratios of dyes and NPs which correspond to

the number of dye molecules *per* NP $N_{Dye/NP} = c_{Dye}/c_{NP}$ were found to be 5.48, 5.61, and 7.66 for the Fe_3O_4 PMA-Dy605, Fe_3O_4 PMA-Furf-Cy5.5, and Fe_3O_4 PMA-Prop-Coumarin NPs, respectively, see Table S1. This confirms that a similar number of dyes *per* NP was attached for all cases. Errors in the absolute numbers may in particular arise from uncertainties in the determination of ε_{NP}.

A

Sample	A_{450}	A_{600}	c_{NP} [nM]	c_{Dye} [nM]	c_{Dye}/c_{NP}	I_{625} [a.u.]
Fe_3O_4 PMA-Dy605	0.485	0.360	414.5	2272.7	5.48	$5.60 \cdot 10^6$

B

Sample	A_{450}	A_{673}	c_{NP} [nM]	c_{Dye} [nM]	c_{Dye}/c_{NP}	I_{720} [a.u.]
Fe_3O_4 PMA-Furf-Cy5.5	0.553	0.554	472.6	2650.7	5.61	$4.39 \cdot 10^5$

C

Sample	A_{500}	A_{437}	c_{NP} [nM]	c_{Dye} [nM]	c_{Dye}/c_{NP}	I_{485} [a.u.]
Fe_3O_4 PMA-Prop-Coumarin	0.195	0.540	318.1	2435.9	7.66	$2.16 \cdot 10^6$

Table S1. *Quantification of the number of dye molecules linked per NP in the different samples: (A) Fe_3O_4 PMA-Dy605 NPs, (B) Fe_3O_4 PMA-Furf-Cy5.5 NPs, and (C) Fe_3O_4 PMA-Prop-Coumarin NPs. The table comprises the recorded absorption values A at two wavelengths, the concentrations of the NPs c_{NP} and the dyes c_{Dye}, and the ratio of dyes per NP and the fluorescence intensity of the dyes.*

As already mentioned, the absorbance at 450 nm A_{450} was used for Fe_3O_4 NPs conjugated with Dy605 and Cy5.5 since corresponding free dyes do not have any absorbance at this wavelength. For Fe_3O_4-coumarin NPs, absorbance at 500 nm A_{500} was collected instead of 450 nm, as coumarin has ignorable absorbance at 500 nm. Besides, to calculate the concentration of coumarin, the absorbance at 437 nm was collected. As Fe_3O_4 NPs also has absorbance peak at 437 nm, after subtracting the contribution from NPs, the concentration of coumarin was calculated. The absorbance at 500 nm and 437 nm ($A_{500} = 0.195$, $A_{437} = 0.54$) was from the absorbance spectra in Figure S11 A. The molar extinction coefficients of Fe_3O_4 at 437 nm and 500 nm was determined based on the absorption spectrum and the molar extinction at at 450 nm (Figure S11 C and S11 D, $\varepsilon_{NP}(500) = 6.13 \times 10^5$ $M^{-1}cm^{-1}$, $\varepsilon_{NP}(437) = 1.40 \times 10^6$ $M^{-1}cm^{-1}$) . The concentration of NPs was calculated as $c_{NP} = A_{500}/(\varepsilon_{NP}(500) \cdot l) = 318.1$ nM. The absorbance at 437 nm part from the NPs and from the coumarin was determined as $A^1_{437} = c_{NP} \cdot \varepsilon_{NP}(437) \cdot l = 0.445$ and $A^2_{437} = A_{437} - A^1_{437} = 0.0.095$, respectively. Finally, the concentration of coumarin was calculated as $c_{Coumarin} = A^2_{437}/(\varepsilon_{Dye}(437) \cdot l) = 2435.9$

nM ($\varepsilon_{Dye}(437) = 39000\ M^{-1}cm^{-1}$). The ratio of conjugated Cy5.5 *per* NP was c_{Dye}/c_{NP} = 7.66, and the fluorescence intensity at the peak at 720 nm was 2.16×10^6.

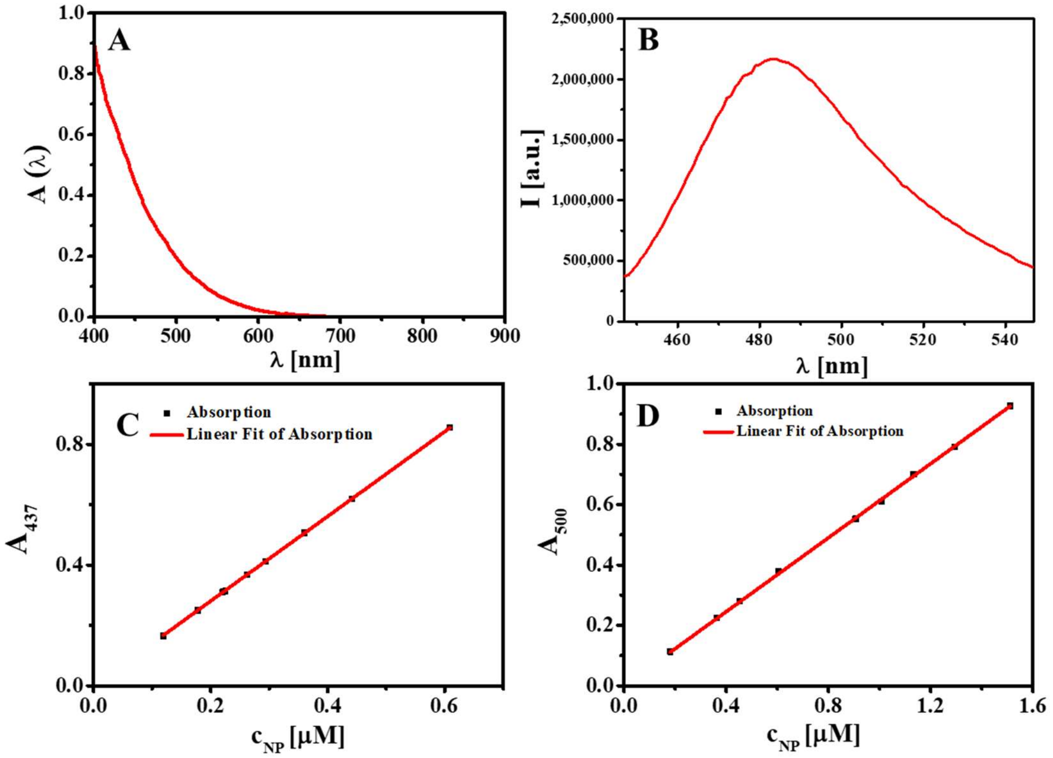

Figure S11. *(A, B) UV-vis absorption spectra A(λ) and fluorescence spectra I(λ) of Fe_3O_4 NPs conjugated with coumarin as recorded in MilliQ water. (C, D) Absorption at 437 and 500 nm of this sample in a cuvette of path length l= 1 cm, plotted versus the NP concentration, c_{NP}, which was used to determine the extinction coefficients at two different wavelengths.*

In Fe_3O_4 PMA-Dy605 samples, the absorbance at 450 nm (A_{450} = 0.485, λ = 450 nm is the wavelength used to determine the NPs concentration) and the absorbance at 600 nm (A_{600} = 0.36, λ = 600 is the wavelength of maximum dye absorbance) were recorded and are shown in Figure S12 A. Based on the molar extinction coefficient of Fe_3O_4 NPs at 450 nm as described before (ε_{NP} (450) = $1.17\times10^6\ M^{-1}cm^{-1}$), and the molar extinction coefficient value of Dy605 provided by the supplier at the absorbance peak at 600 nm ($\varepsilon_{Dy605}(600) = 110000\ M^{-1}cm^{-1}$), applying the Beer-Lambert's law, concentrations of NPs, Dy605, and the ratio of dyes *per* NP were calculated (c_{NP} = 414.5 nM, c_{Dye} = 2272.7 nM, c_{Dye}/c_{NP} = 5.48). Apart from the absorbance spectra and calculated values above, fluorescence spectra and intensity at the emission maximum at 625 nm (I_{625} = $5.6\text{x}10^6$ [a.u.]) are all illustrated in Figure 12 B.

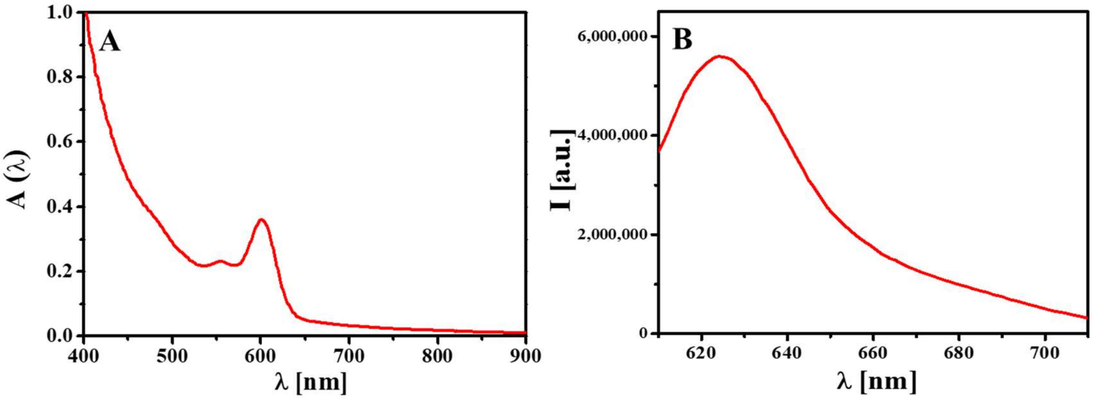

Figure S12. (A) *UV-vis absorption spectra A(λ) and (B) fluorescence spectra I(λ) of* Fe_3O_4 PMA-Dy605 NPs *as recorded in MilliQ water.*

To determine the dye conjugation of Fe_3O_4 PMA-Furf-Cy5.5 quantitatively, a similar calculation process was performed. The corresponding spectra are shown in Figure S13. With the absorbance at 450 nm and at 684 nm (A_{450} = 0.553, A_{673} = 0.554), the molar extinction coefficient of Fe_3O_4 NPs ($\varepsilon_{NP}(450) = 1.17\times10^6$ $M^{-1}cm^{-1}$), and the molar extinction coefficient value of Cy5.5 at 684 nm ($\varepsilon_{Cy5.5}(673)$ = 209000 $M^{-1}cm^{-1}$), the final concentration of the NPs and Cy5.5 was calculated as $c_{NP} = A_{450}/(\varepsilon_{NP}(450)\cdot l)$ = 472.6 nM, $c_{Dye} = A_{673}/(\varepsilon_{Cy5.5}(684)\cdot l)$ = 2650.7 nM. Thus, the ratio of conjugated Cy5.5 *per* NP was c_{Dye}/c_{NP} = 5.61, and the fluorescence intensity at the peak of 720 nm was $4.39\text{x}10^5$.

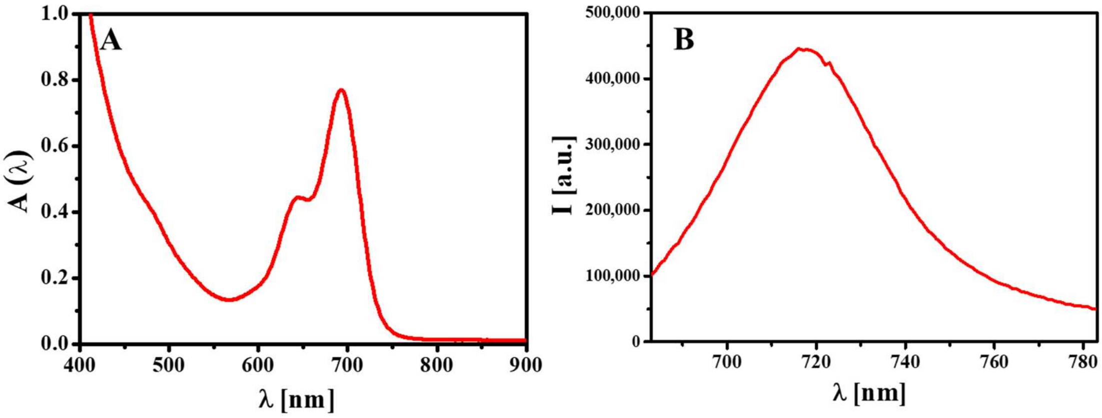

Figure S13. (A) *UV-vis absorption spectra A(λ) and (B) fluorescence spectra I(λ) of Fe_3O_4 PMA-Prop-Cy5.5 NPs as recorded in MilliQ water.*

III. Enzyme-induced degradation of the polymer shell of dye conjugated Fe_3O_4 NPs

III.1 NP incubation with enzymes

The enzyme solutions had approximately the same pH as the buffer in which the enzymes were dissolved, *i.e.* the enzymes did not change the pH, see Figure S14.

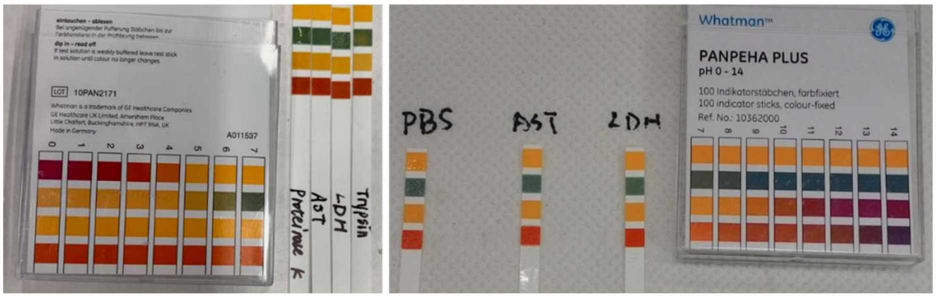

Figure S14. *The pH of the solutions with enzyme dissolved in buffer (Proteinase K, AST, LDH, Trypsin; AST and LDH in PBS) was similar to the pH of the plain buffer solution (PBS).*

The dye-conjugated Fe_3O_4 NPs were incubated with fetal bovine serum (FBS) and 6 different enzymes. This was done by diluting 5 µL (c_{NP} = 0.5 µM) concentrated Fe_3O_4 NPs and 5 µL (c_{FBS} = 100%) FBS or enzyme with 490 µL phosphate buffered saline (PBS) solution at pH 7.4 to get a final mixture volume of 500 µL. Thus, the final NP and FBS concentrations were c_{NP} = 5 nM and c_{FBS}= 1%, respectively. These samples were incubated for 24 h at 37 °C. Fe_3O_4 PMA NPs in PBS without the enzyme and only enzyme without the NPs served as two controls. After the incubation, the fluorescence intensities I_0 of these samples were measured with a Fluorolog-3 (model FL3-22) from Horiba.

As next step the samples were filtered with a centrifugal filter (500 µL, Amicon Ultra, 100 kDa MWCO) for 10 minutes at 9000 rpm, whereby the NPs are retained, and dye which got detached due to enzymatic digestion is in the eluate [5]. Filtration was performed to leave as litte volume retained as possible. The eluent from the bottom of the filter was collected and adjusted again with PBS to 500 µL, in order to keep the same volume as the original solution. The fluorescence intensity I_1 of the eluent was recorded. Apart from FBS (1%), Trypsin (0.01%), CAT G (10 U/mL), LDH (10 U/mL), ACHE (10 U/mL), AST (5 U/L), and Proteinase K (10 U/mL) were also incubated with the different NPs. Additionally, the impact of enzyme concentration on the degradation efficiency was studied.

III.2 Fluorescence spectra of NPs incubated with enzyme (I_0)

In Figure S15, the fluorescence spectra of Fe_3O_4 PMA-Prop-Coumarin, Fe_3O_4 PMA-Dy605 and Fe_3O_4 PMA-Furf-Cy5.5 NPs after incubation with enzymes are presented, together with the spectra of the enzymes themselves. Compared with the fluorescence intensity of dye conjugated NPs, the enzymes themselves shown negligible fluorescence (Figure S15 A, C, E). In the emission spectra of the dye conjugated NPs (Figure S15 B, D, F), the fluorescence intensity of various samples incubated with enzymes were higher than the ones incubated in PBS without enzymes, which may be caused by the removal of distance quenching caused by underlying Fe_3O_4 NPs.

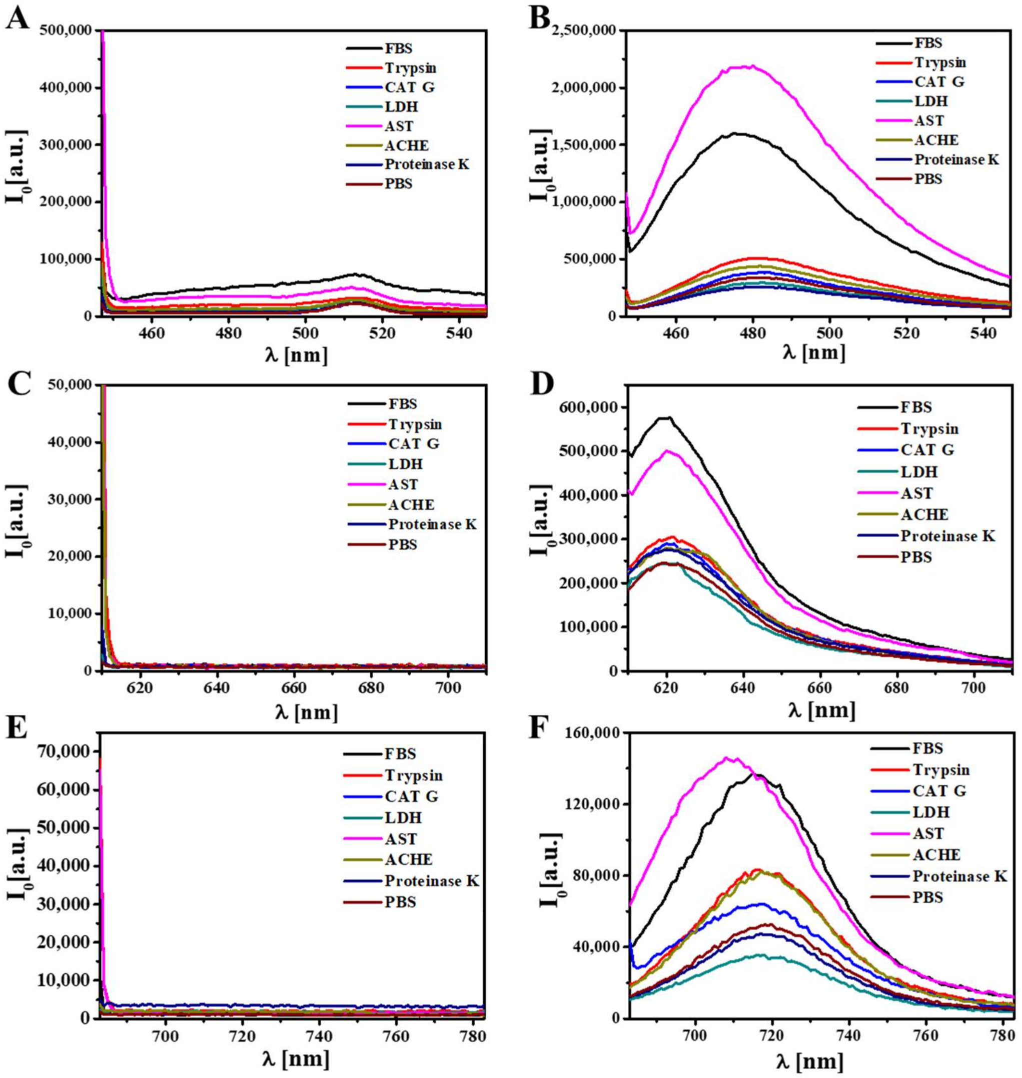

Figure S15. *Emission spectra (I_0) of different enzymes (control) on the left side at the emission wavelength range of (A) Coumarin, (C) Dy605, and (E) Cy5.5. Emission spectra I_0 of the NP samples with added enzymes: (B) Fe_3O_4 PMA-Prop-Coumarin NPs, (D) Fe_3O_4 PMA-Dy605, and (F) Fe_3O_4 PMA-Furf-Cy5.5 on the right side.*

III.3 Fluorescence spectra of released dye in the eluate (I_1)

The emission spectra of the eluates of Fe_3O_4 PMA-Prop-Coumarin, Fe_3O_4 PMA-Dy605, and Fe_3O_4 PMA-Furf-Cy5.5 NPs are presented in Figure S16 B, D, F, together with the emission spectra of controls with enzymes only in Figure S16 A, C, E. The results show detachment of all three dyes (*i.e.* major fluorescence in the eluate), majorly in the cases of FBS and AST. Trypsin shows moderate degradation capacity.

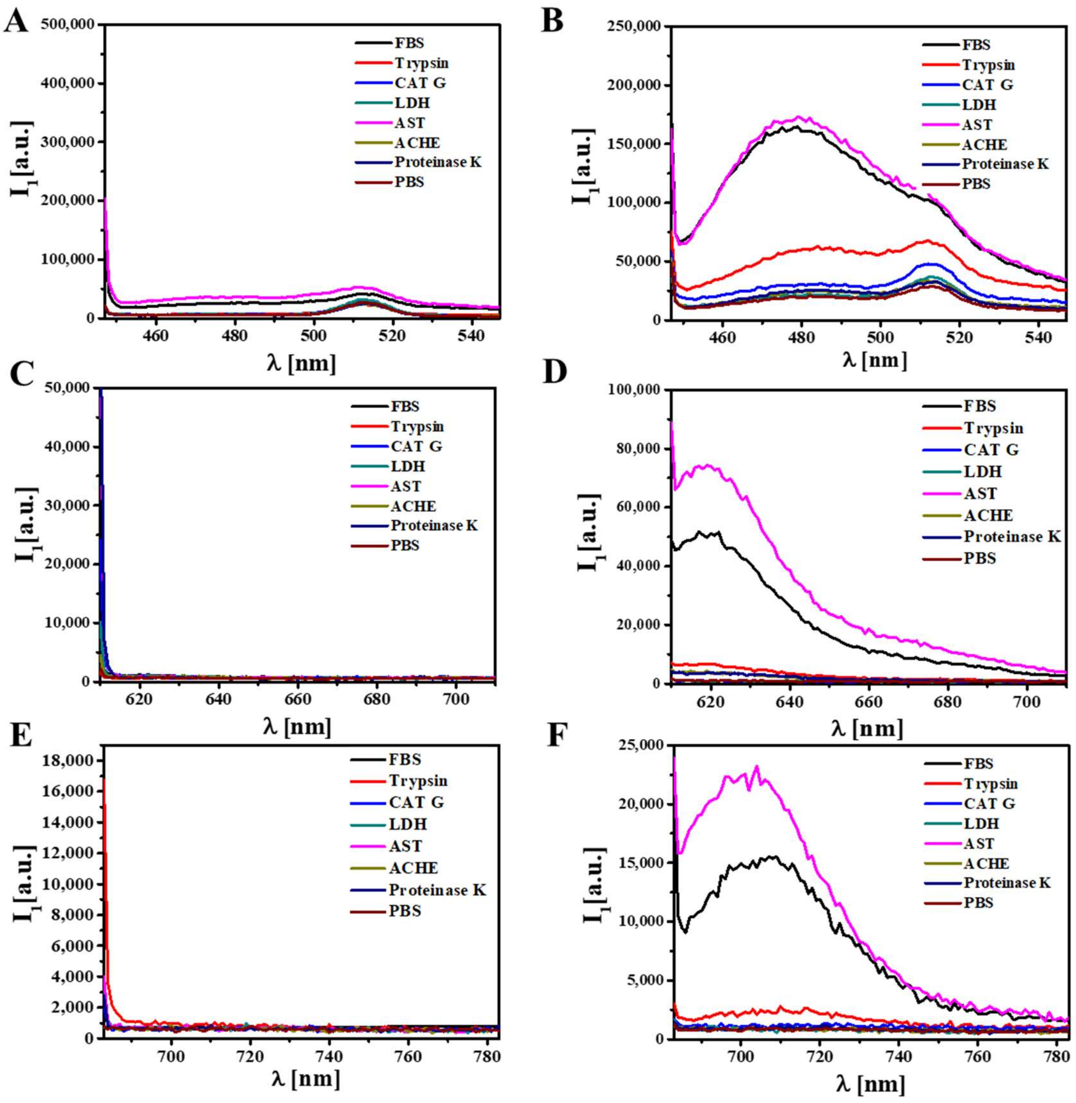

Figure S16. *Emission spectra (I_1) of different enzymes (control) on the left side at the emission wavelength range of (A) Coumarin, (C) Dy605, and (E) Cy5.5. Emission spectra I_0 of NP samples with enzymes: (B) Fe_3O_4 PMA-Prop-Coumarin NPs (D) Fe_3O_4 PMA-Dy605, and (F) Fe_3O_4 PMA-Furf-Cy5.5 on the right side.*

III.4 Dependence on enzyme concentration

The impact of enzyme concentration was studied for the degradation, which shows a linearly increasing effect in the I_1 values for AST and FBS in all three dye-conjugated NP cases. The intensities are plotted in Figure 3 of the main manuscript and one set of emission spectra for FBS is shown in Figure S17. From these I_0 and I_1 fluorescence spectra corresponding to the incubation Fe_3O_4 PMA-Prop-Coumarin NPs (Figure S17 A, D), Fe_3O_4 PMA-Dy605 (Figure S17 B, E), and Fe_3O_4 PMA-Furf-Cy5.5 (Figure S17 C, F) with enzymes, the amount of cleaved dye was increased with the increasing FBS concentration in all cases.

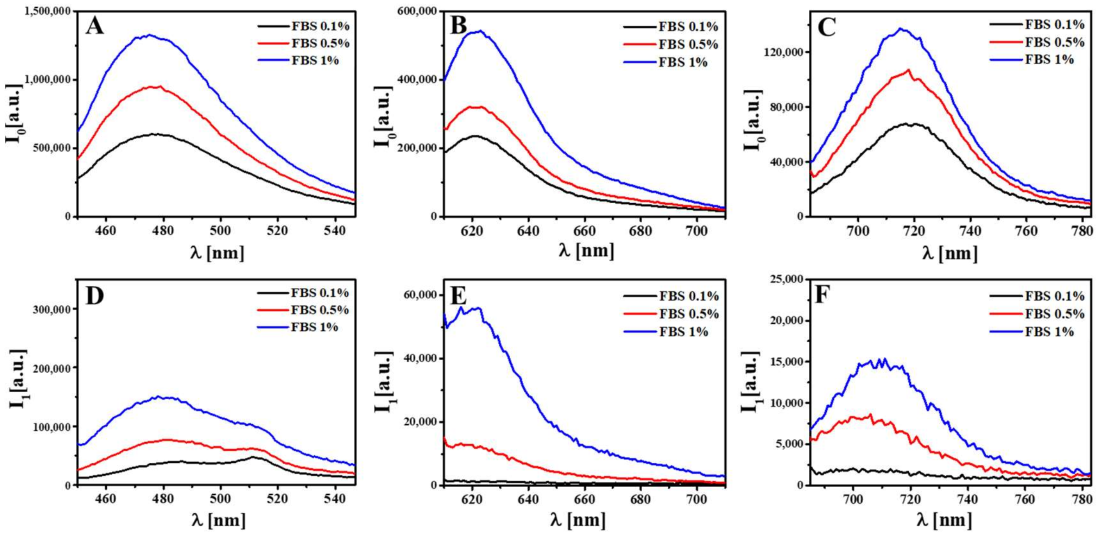

Figure S17. *Effect of enzyme concentration for the case of FBS in the incubation experiment. Emission spectra corresponding to $I_0(\lambda)$ (top row, A-C) and $I_1(\lambda)$ (bottom row, D-F) of (A, D) Fe_3O_4 PMA-Prop-Coumarin, (B, E) Fe_3O_4 PMA-Dy605, and (C, F) Fe_3O_4 PMA-Furf-Cy5.5 are shown.*

In Figures S18-S20, the effects of enzyme concentration (trypsin, CATG, LDH, ACHE and Proteinase K) on I_1 are presented. The Trypsin data also show a linear relation, but with a lower slope. For the other cases, the intensity did not change much with the concentration, which may because of the relatively slighter capability of degradation.

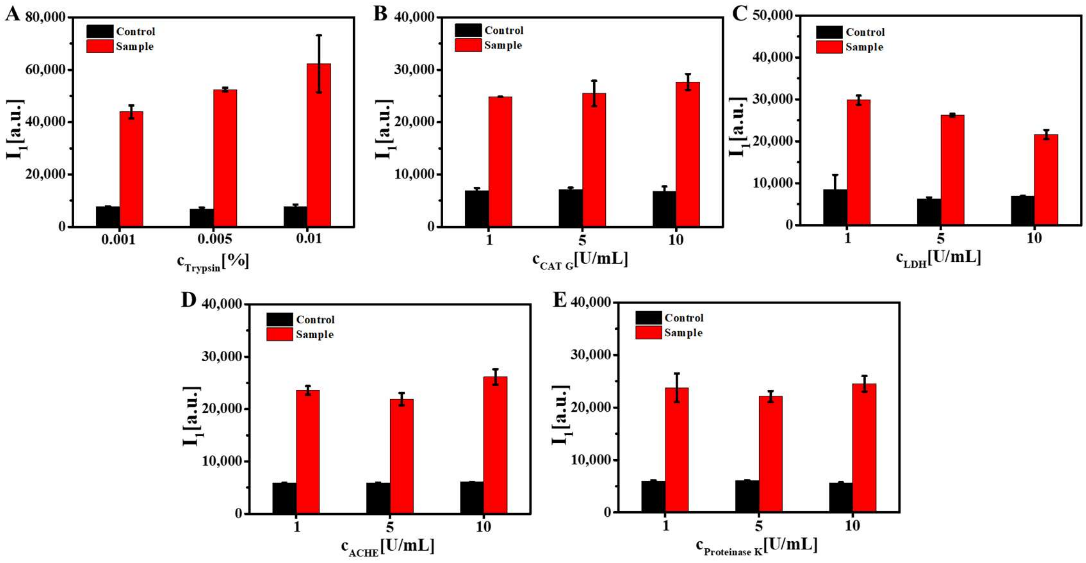

Figure S18. *Effect of enzyme concentrations in the incubation experiment for* Fe_3O_4 *PMA-Prop-Coumarin NPs. The emission intensities* I_1 *are plotted for enzyme degradation experiments with (A) trypsin, (B) CAT G, (C) LDH, (D) ACHE, and (E) Proteinase K at different enzyme concentrations. The controls represent the same experiment without the presence of NPs, i.e. just the enzymes (black trace).*

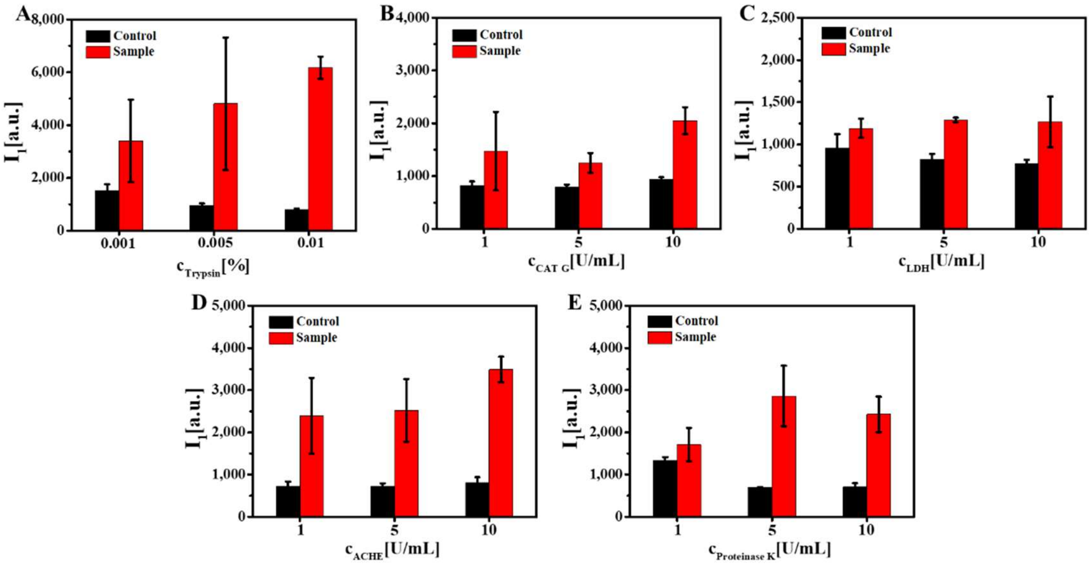

Figure S19. *Effect of enzyme concentrations in the incubation experiment for* Fe_3O_4 *PMA-Dy605 NPs. The emission intensities* I_1 *are plotted for enzyme degradation experiments with (A) trypsin, (B) CAT G, (C) LDH, (D) ACHE, and (E) Proteinase K at different enzyme concentrations. The controls represent the same experiment without the presence of NPs, i.e. just the enzymes (black trace).*

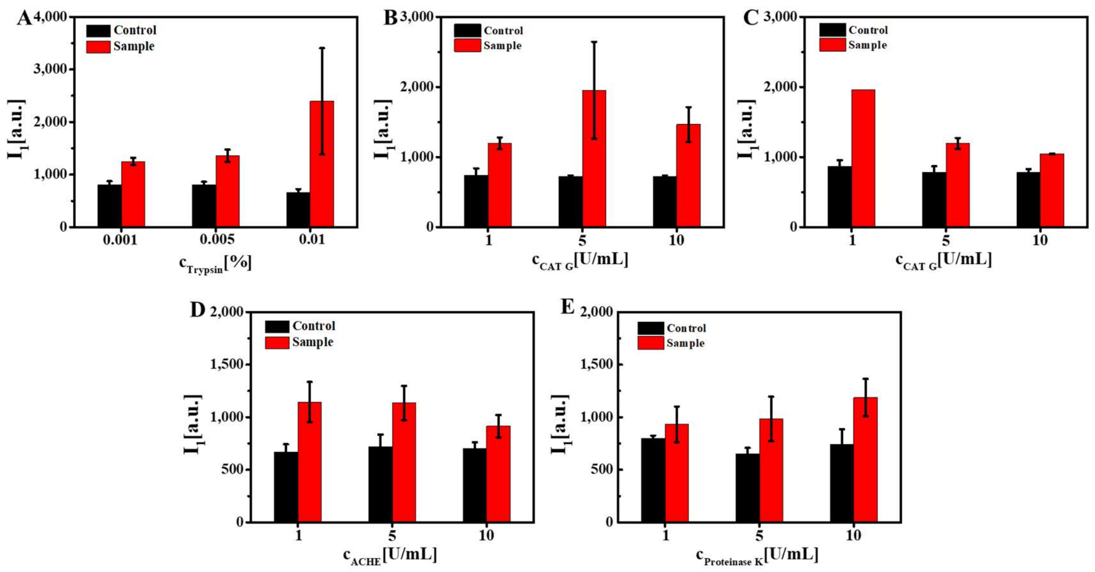

Figure S20. *Effect of enzyme concentrations in the incubation experiment for Fe_3O_4 PMA-Furf-Cy5.5 NPs. The emission intensities I_1 are plotted for enzyme degradation experiments with (A) trypsin, (B) CAT G, (C) LDH, (D) ACHE, and (E) Proteinase K at different enzyme concentrations. The controls represent the same experiment without the presence of NPs, i.e. just the enzymes (black trace).*

The relation of I_1/I_0 values with different enzyme concentrations in samples of Fe_3O_4 PMA-Prop-Coumarin, Fe_3O_4 PMA-Dy605, and Fe_3O_4 PMA-Furf-Cy5.5 NPs are shown in Figures S21-S23. As argued in the main manuscript, the I_1/I_0 values quantify the cleavage percentage. In the FBS group, the I_1/I_0 values rose linearly with the FBS concentration. The percentage of cleaved dye in trypsin showed a mild increase with enzyme concentration. In the case of AST, only Fe_3O_4 PMA-Prop-Coumarin NPs were unaffected by the enzyme concentration, while in Fe_3O_4 PMA-Dy605 and Fe_3O_4 PMA-Furf-Cy5.5 NPs, the percentages of cleaved dye grew evidently with enzyme concentration. For the other groups, the changes of I_1/I_0 values with enzyme concentration were not so obvious.

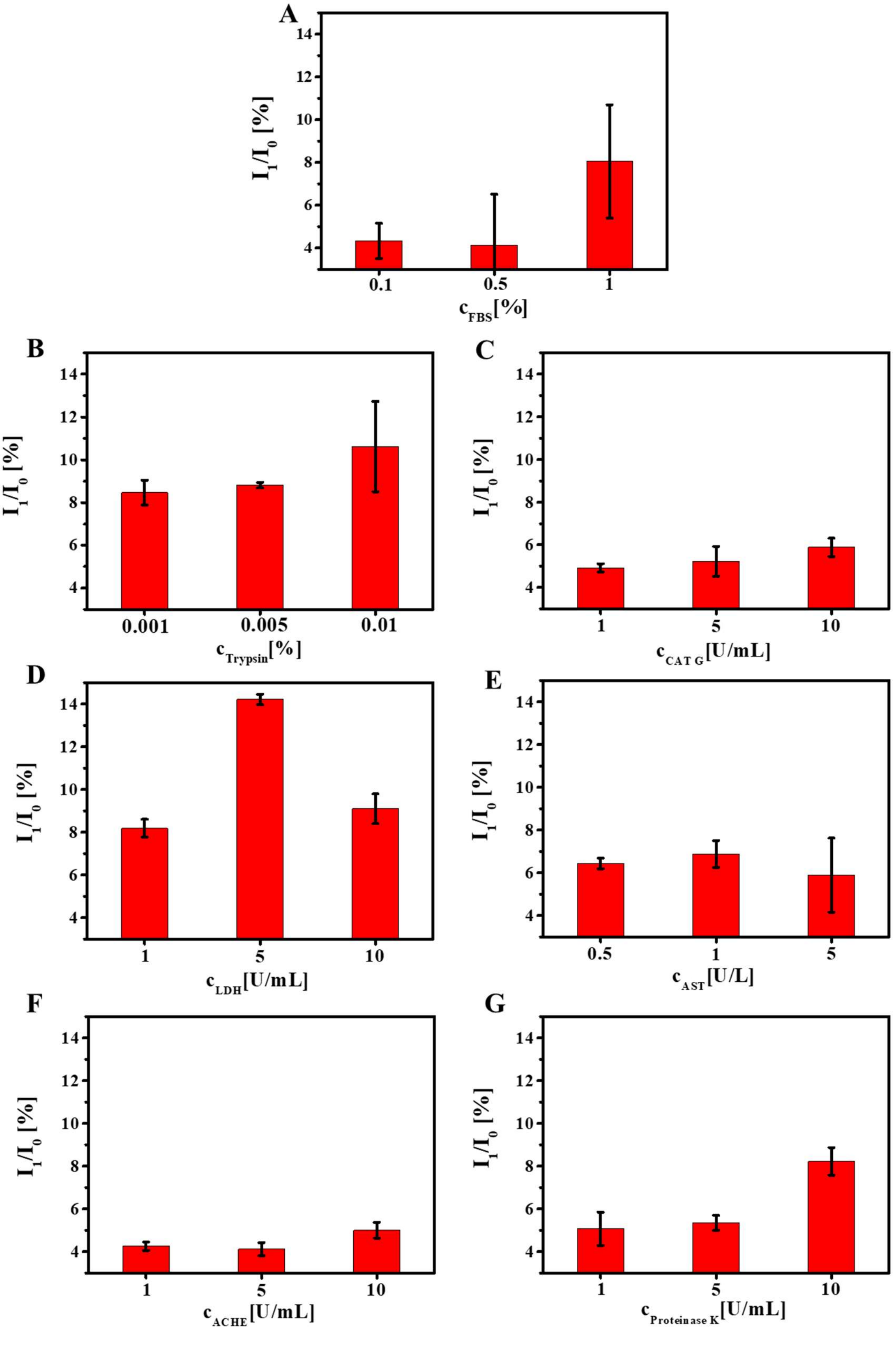

Figure S21. *Effect of concentration of enzymes on the I_1/I_0 value for Fe_3O_4 PMA-Prop-Coumarin. The I_1/I_0 value are presented for different enzymes: (A) FBS, (B) Trypsin, (C) CATG, (D) LDH, (E) AST, (F) ACHE, and (G) Proteinase K.*

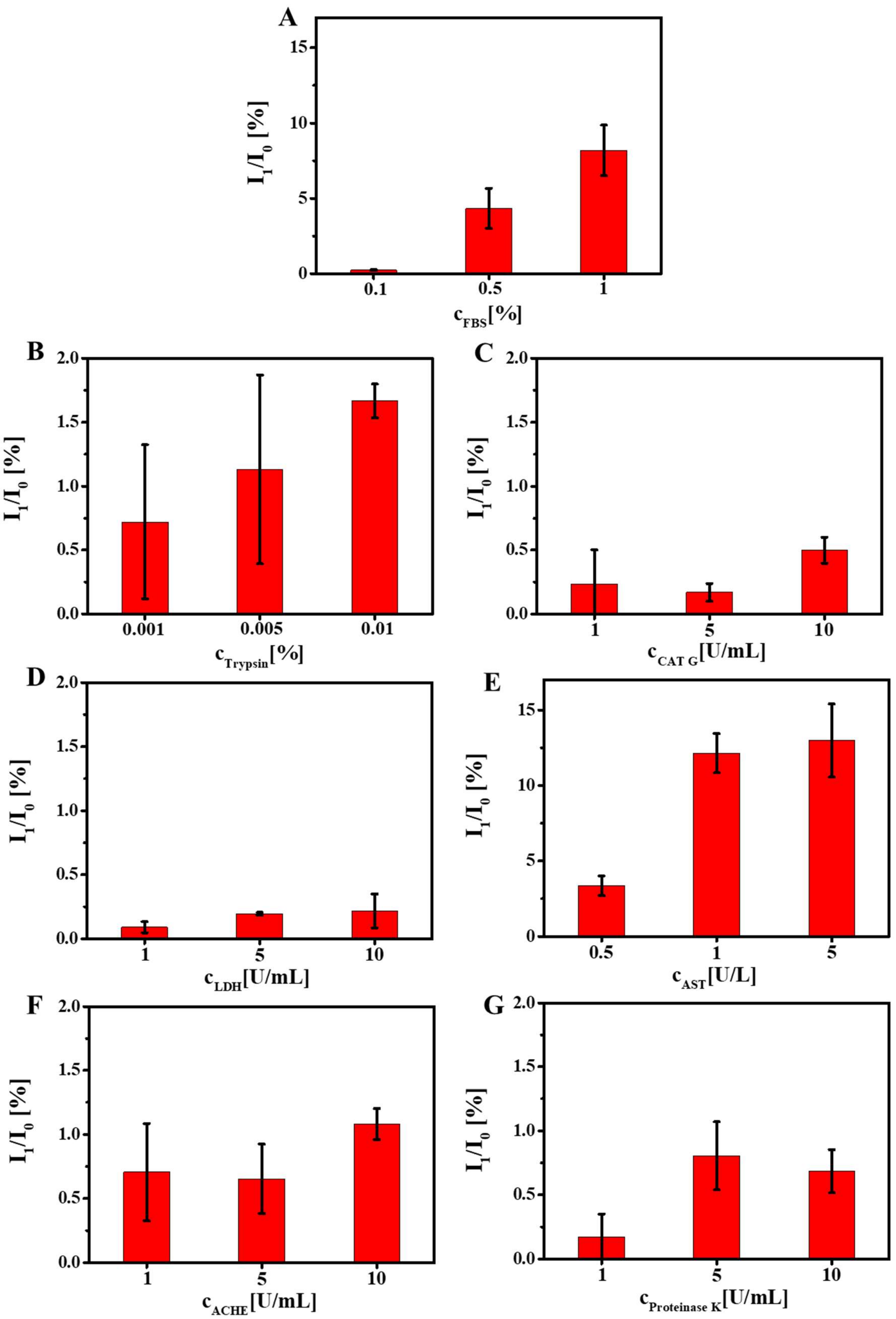

Figure S22. *Effect of concentration of enzymes on the I_1/I_0 value for Fe_3O_4 PMA-Dy605. The I_1/I_0 value are presented for different enzymes: (A) FBS, (B) Trypsin, (C) CATG, (D) LDH, (E) AST, (F) ACHE, and (G) Proteinase K.*

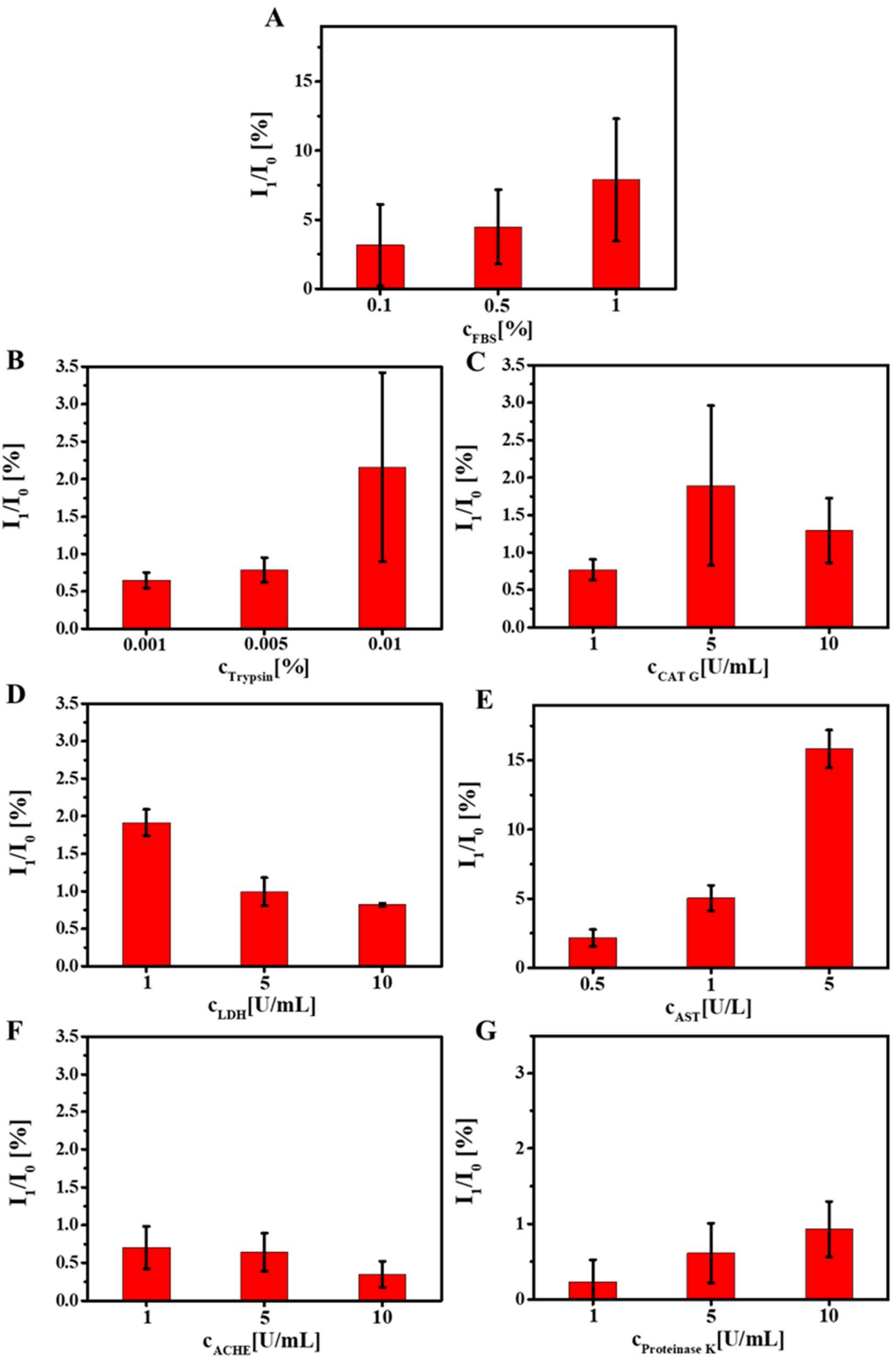

Figure S23. *Effect of concentration of enzymes on the I_1/I_0 value for Fe_3O_4 PMA-Furf-Cy5.5. The I_1/I_0 value are presented for different enzymes: (A) FBS, (B) Trypsin, (C) CATG, (D) LDH, (E) AST, (F) ACHE, and (G) Proteinase K.*

III.5 Control experiments

Additional control experiments were carried out to understand the degradation in more detail. The iron content was measured for the lower part (*i.e.* the eluate) as well as upper part (*i.e.* the retained NPs) of the centrifuged samples after enzyme incubation (for the case of FBS). This was to check whether the fluorescence intensities of the lower part are due to leaking of intact NPs through the centrifuge filter or not. An Agilent ICP-MS 7500cs inductively coupled plasma-mass spectrometry instrument was used for the analysis. Both the lower and upper part was volume-adjusted to 500 µL. Then, 50 µL of the solution was taken from each part and digested with 150 µL of freshly prepared aqua-regia. The mixture was kept on a shaker for 2 h. The digested samples were then further diluted by 2% of HCl and were then used for the ICP-MS analysis. The corresponding data are presented in Figure S24 A which shows the negligible amounts of iron in the lower part, confirming that the degradation is only from the polymer-shell part, but not from the inorganic Fe_3O_4 core. For further confirmation, the absorption spectrum of the lower part was collected and compared to the one of free dye and dye-conjugated NPs. The absorption spectrum of the eluate shows the features of the dye, but no absorbance features corresponding to the NPs near 400-450 nm were noticed (Figure S24 B).

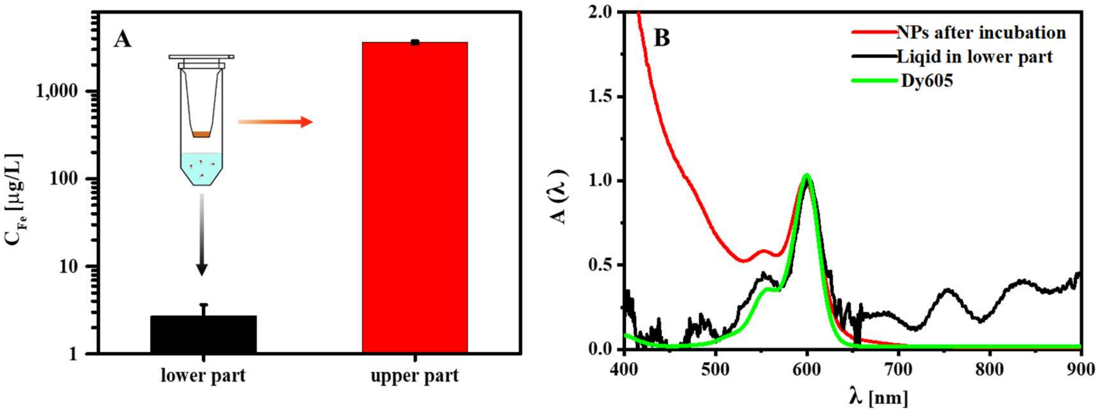

Figure S24. *(A) The iron content of Fe_3O_4 NPs from the upper and lower part of the centrifuge filter was determined by ICP-MS. (B) Normalized absorption spectra $A(\lambda)$ of Fe_3O_4 NPs after incubation with FBS (red trace), the eluent (black trace) and the spectrum of free dye (green trace).*

Control experiments with only dye were also performed in Figure S25. In this case, Dy605 dye was incubated with FBS and then I_1 and I_0 were collected similarly as mentioned in Chapter III.1 at different concentration of FBS. In this case, the total amount of dyes was not recovered in the eluate (though the free dyes should all cross

the filter membrane), which suggests that some of the disconnected dye molecules remain adsorbed to the enzyme surface and remain in the upper part or adsorbed within the filter membranes. Thus, quantitative degradation efficiency analysis is complicated for the case of FBS.

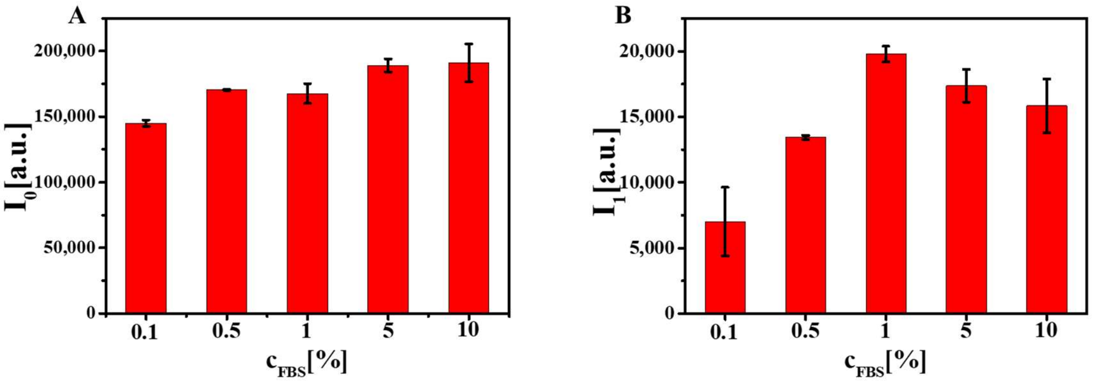

Figure S25. *Control experiment with free dye.* $I_0(\lambda)$ *(A) and* $I_1(\lambda)$ *(B) of the free dye Dy605 (0.1 nM) after incubation with FBS.*

IV References

1. Sun, R. H.; Iribarren, P.; Zhang, N.; Zhou, Y.; Gong, W. H.; Cho, E. H.; Lockett, S.; Chertov, O.; Bednar, F.; Rogers, T. J.; Oppenheim, J. J.; Wang, J. M., Identification of neutrophil granule protein cathepsin G as a novel chemotactic agonist for the G protein-coupled formyl peptide receptor. *J. Immunol.* **2004,** 173, (1), 428-436.
2. Lin, C.-A. J.; Sperling, R. A.; Li, J. K.; Yang, T.-Y.; Li, P.-Y.; Zanella, M.; Chang, W. H.; Parak, W. J., Design of an Amphiphilic Polymer for Nanoparticle Coating and Functionalization. *Small* **2008,** 4, (3), 334-341.
3. Zhang, F.; Lees, E.; Amin, F.; Rivera_Gil, P.; Yang, F.; Mulvaney, P.; Parak, W. J., Polymer-Coated Nanoparticles: A Universal Tool for Biolabelling Experiments. *Small* **2011,** 7, 3113-3127.
4. Sperling, R. A.; Parak, W. J., Surface modification, functionalization and bioconjugation of colloidal inorganic nanoparticles. *Philosophical Transactions of the Royal Society A - Mathematical Physical and Engineering Science* **2010,** 368, (1915), 1333-1383.
5. Hühn, J.; Carrillo-Carrion, C.; Soliman, M. G.; Pfeiffer, C.; Valdeperez, D.; Masood, A.; Chakraborty, I.; Zhu, L.; Gallego, M.; Zhao, Y.; Carril, M.; Feliu, N.; Escudero, A.; Alkilany, A. M.; Pelaz, B.; Pino, P. d.; Parak, W. J., Selected Standard Protocols for the Synthesis, Phase Transfer, and Characterization of Inorganic Colloidal Nanoparticles. *Chem. Mater.* **2017,** 29, 399−461.
6. Ali, Z.; Abbasi, A. Z.; Zhang, F.; Arosio, P.; Lascialfari, A.; Casula, M. F.; Wenk, A.; Kreyling, W.; Plapper, R.; Seidel, M.; Niessner, R.; Knoll, J.; Seubert, A.; Parak, W. J., Multifunctional Nanoparticles for Dual Imaging. *Anal. Chem.* **2011,** 83, (8), 2877-2882.
7. Kreyling, W. G.; Abdelmonem, A. M.; Ali, Z.; Alves, F.; Geiser, M.; Haberl, N.; Hartmann, R.; Hirn, S.; de Aberasturi, D. J.; Kantner, K.; Khadem-Saba, G.; Montenegro, J. M.; Rejman, J.; Rojo, T.; de Larramendi, I. R.; Ufartes, R.; Wenk, A.; Parak, W. J., In vivo integrity of polymer-coated gold nanoparticles. *Nature Nanotechnol* **2015,** 10, (7), 619-623.
8. Menendez-Miranda, M.; Costa-Fernandez, J. M.; Encinar, J. R.; Parak, W. J.; Carrillo-Carrion, C., Determination of the ratio of fluorophore/nanoparticle for fluorescence-labelled nanoparticles. *Analyst* **2016,** 141, (4), 1266-1272.
9. Fernández-Argüelles, M. T.; Yakovlev, A.; Sperling, R. A.; Luccardini, C.; Gaillard, S.; Medel, A. S.; Mallet, J.-M.; Brochon, J.-C.; Feltz, A.; Oheim, M.; Parak, W. J., Synthesis and Characterization of Polymer-Coated Quantum Dots with Integrated Acceptor Dyes as FRET-based Nanoprobes. *Nano Lett.* **2007,** 7, (9), 2613-2617.
10. Tang W; ML, B., ŧ Click ヤ reactions: a versatile toolbox for the synthesis of peptide-conjugates. *Chem. Soc. Rev.* **2014,** 43, 7013-7039.
11. Kolb, H. C.; Finn, M. G.; Sharpless, K. B., Click Chemistry: Diverse Chemical Function from a Few Good Reactions. *Angew. Chem., Int. Ed.* **2001,** 40, (11), 2004-2021.

12. Liang, L. Y.; Astruc, D., The copper(I)-catalyzed alkyne-azide cycloaddition (CuAAC) "click" reaction and its applications. An overview. *Coord. Chem. Rev.* **2011,** 255, (23-24), 2933-2945.
13. Nicolaou, K. C.; Snyder, S. A.; Montagnon, T.; Vassilikogiannakis, G., The Diels-Alder reaction in total synthesis. *Angew. Chem.-Int. Edit.* **2002,** 41, (10), 1668-1698.
14. Castro, V.; Rodriguez, H.; Albericio, F., CuAAC: An Efficient Click Chemistry Reaction on Solid Phase. *ACS combinatorial science* **2016,** 18, (1), 1-14.
15. Woodward, R. B.; Katz, T. J., THE MECHANISM OF THE DIELS-ALDER REACTION. *Tetrahedron* **1959,** 5, (1), 70-89.
16. Wang, C.; Yan, Q.; Liu, H. B.; Zhou, X. H.; Xiao, S. J., Different EDC/NHS Activation Mechanisms between PAA and PMAA Brushes and the Following Amidation Reactions. *Langmuir* **2011,** 27, (19), 12058-12068.
17. Fischer, M. J. E., Amine coupling through EDC/NHS: a practical approach. *Methods in molecular biology (Clifton, N.J.)* **2010,** 627, 55-73.
18. Pelaz, B.; Del Pino, P.; Maffre, P.; Hartmann, R.; Gallego, M.; Rivera-Fernandez, S.; de la Fuente, J. M.; Nienhaus, G. U.; Parak, W. J., Surface Functionalization of Nanoparticles with Polyethylene Glycol: Effects on Protein Adsorption and Cellular Uptake. *ACS Nano* **2015,** 9, (7), 6996-7008.
19. Lutz, J. F., 1,3-dipolar cycloadditions of azides and alkynes: A universal ligation tool in polymer and materials science. *Angew. Chem.-Int. Edit.* **2007,** 46, (7), 1018-1025.
20. Muller, J. G.; Anni, M.; Scherf, U.; Lupton, J. M.; Feldmann, J., Vibrational fluorescence spectroscopy of single conjugated polymer molecules. *Physical Review B* **2004,** 70, (3).
21. Jang, E. S.; Lee, S. Y.; Cha, E. J.; Sun, I. C.; Kwon, I. C.; Kim, D.; Kim, Y. I.; Kim, K.; Ahn, C. H., Fluorescent Dye Labeled Iron Oxide/Silica Core/Shell Nanoparticle as a Multimodal Imaging Probe. *Pharm. Res.* **2014,** 31, (12), 3371-3378.
